# Ancestral retrovirus envelope protein ERVWE1 upregulates circ_0001810, a potential biomarker for schizophrenia, and induces neuronal mitochondrial dysfunction via activating AK2

**DOI:** 10.1186/s13578-024-01318-1

**Published:** 2024-11-14

**Authors:** Wenshi Li, Xing Xue, Xuhang Li, Xiulin Wu, Ping Zhou, Yaru Xia, Jiahang Zhang, Mengqi Zhang, Fan Zhu

**Affiliations:** State Key Laboratory of Virology, Department of Medical Microbiology, School of Basic Medical Science, Wuhan University, Wuhan, 430071 China

**Keywords:** ERVWE1, Circular RNAs, AK2, Mitochondrial depolarization, Mitochondrial dynamics, Schizophrenia

## Abstract

**Background:**

Increasingly studies highlight the crucial role of the ancestral retrovirus envelope protein ERVWE1 in the pathogenic mechanisms of schizophrenia, a severe psychiatric disorder affecting approximately 1% of the global population. Recent studies also underscore the significance of circular RNAs (circRNAs), crucial for neurogenesis and synaptogenesis, in maintaining neuronal functions. However, the precise relationship between ERVWE1 and circRNAs in the etiology of schizophrenia remains elusive.

**Results:**

This study observed elevated levels of hsa_circ_0001810 (circ_0001810) in the blood samples of schizophrenia patients, displaying a significant positive correlation with ERVWE1 expression. Interestingly, in vivo studies demonstrated that ERVWE1 upregulated circ_0001810 in neuronal cells. Circ_0001810, acting as a competing endogenous RNA (ceRNA), bound to miR-1197 and facilitated the release of adenylate kinase 2 (AK2). The bioinformatics analysis of the schizophrenia datasets revealed increased levels of AK2 and enrichment of mitochondrial dynamics. Notably, miR-1197 was reduced in schizophrenia patients, while AK2 levels were increased. Additionally, AK2 showed positive correlations with ERVWE1 and circ_0001810. Further studies demonstrated that AK2 led to mitochondrial dysfunction, characterized by loss of intracellular ATP, mitochondrial depolarization, and disruption of mitochondrial dynamics. Our comprehensive investigation suggested that ERVWE1 influenced ATP levels, promoted mitochondrial depolarization, and disrupted mitochondrial dynamics through the circ_0001810/AK2 pathway.

**Conclusions:**

Circ_0001810 and AK2 were increased in schizophrenia and positively correlated with ERVWE1. Importantly, ERVWE1 triggered mitochondrial dysfunction through circ_0001810/miR-1197/AK2 pathway. Recent focus on the impact of mitochondrial dynamics on schizophrenia development had led to our discovery of a novel mechanism by which ERVWE1 contributed to the etiology of schizophrenia, particularly through mitochondrial dynamics. Moreover, these findings collectively proposed that circ_0001810 might serve as a potential blood-based biomarker for schizophrenia. Consistent with our previous theories, ERVWE1 is increasingly recognized as a promising therapeutic target for schizophrenia.

**Supplementary Information:**

The online version contains supplementary material available at 10.1186/s13578-024-01318-1.

## Introduction

Human endogenous retroviruses (HERVs), integrated into human DNA millions of years ago, are a remnant of ancient infectious events with transmissible strains [1]. Classified into three categories, HERVs mirror the structure of different virus families: Class I resembles *Gammaretrovirus*, Class II aligns with *Betaretrovirus*, and Class III with *Spumavirus* [2]. It has been further discovered that HERVs can be transcriptionally activated by various factors, including NF-kB [3], external viruses [4], and even common drugs like caffeine and aspirin [5]. Their association with a range of diseases, particularly autoimmune diseases, cancers, and neuropsychiatric disorders [6–8] like schizophrenia [9–11], has been increasingly recognized.

The HERV W family (HERV-W), a Class I transposable elements, is one of the oldest groups of HERVs. ERVWE1, also known as HERV-W env, ERVW-1 or syncytin-1, is an envelope protein derived from HERV-W. It plays a critical role in the formation of syncytiotrophoblast during embryogenesis [12]. Various researches have revealed abnormal expression of ERVWE1 in schizophrenia patients [13, 14], highlighting its potential significance in the development of schizophrenia [7, 15, 16].

Schizophrenia, complex psychiatric disorder, is among the most disabling conditions globally [17]. It disrupts normal brain functions and is diagnosed based on a spectrum of symptoms, including delusions, hallucinations, social withdrawal, and cognitive deficits [18]. Despite various proposed hypotheses about its onset and progression, the incidence and cure rates for schizophrenia have seen little improvement [19–21]. In recent studies, mitochondria, the cellular powerhouses, have come under scrutiny, especially concerning their dynamics in schizophrenia [22]. This focus opens new avenues for understanding and potentially treating this intricate disorder.

Mitochondrial dynamics involve the continuous reshaping of the mitochondrial network within cells, encompassing processes such as organelle fusion and fission and ultrastructural membrane remodeling [23]. Altered expression of proteins involved in these dynamics is increasingly linked to mental disorders [24]. Maintaining a balanced and dynamic mitochondrial network is essential for preserving structural integrity and functionality, particularly for efficient adenosine triphosphate (ATP) generation [25].

In the context of schizophrenia, AK2, an adenylate phosphotransferase located in the intermembrane spaces of mitochondria, emerges as an intriguing subject. Despite its potential relevance, research into AK2's mechanisms in relation to schizophrenia has been surprisingly limited.

CircRNAs have garnered significant attention for their associations with neurological diseases, including schizophrenia [26]. These non-coding RNA molecules, characterized by their covalently closed loop structure without a 5′-terminal cap and a 3′-terminal poly-A tail, were once considered mere byproducts of aberrant splicing [27]. However, recent investigations have unveiled their roles as miRNA sponges, influencing gene translation and performing other regulatory functions [28, 29]. Perturbations in circRNAs have been observed in human fluids, including peripheral blood, suggesting their potential as biomarkers or therapeutic targets in schizophrenia [30, 31]. Furthermore, several researchers have indicated that circRNAs play a role in brain diseases by affecting mitochondrial function [32, 33]. Despite these promising leads, in-depth research into the specific roles and mechanisms of circRNAs in schizophrenia remains scarce, highlighting an area ripe for exploration.

CircRNAs, known for their propensity to interact with miRNAs, play critical roles in variety of cellular processes [28]. MiRNAs, small non-coding RNAs ranging from 19 to 25 nucleotides, regulate gene expression post-transcriptionally by binding to the 3'-UTRs of target mRNA molecules [34]. Recent investigations have focused on the role of miRNAs in mitochondrial dysfunction [35]. In schizophrenia, several miRNAs have been identified through expression profiling studies, highlighting their potential relevance to the disease’s complex and still largely unknown etiology [36]. This underscores the complexity of schizophrenia and the need for continued research into its underlying mechanisms.

This study focuses on two key aspects implicated in the development of schizophrenia: the elevation of ERVWE1 and aberrant mitochondrial dynamics. Surprisingly, the interplay between ERVWE1 elevation and aberrant mitochondrial dynamics has not been extensively explored. We identified a novel circular RNA, circ_0001810, elevated in patients with schizophrenia. Our subsequent research involved a series of experiments to understand how ERVWE1 contributed to ATP depletion, dissipation of the mitochondrial membrane potential (MMP), and the imbalance in mitochondrial dynamics via circ_0001810/miR-1197/AK2 pathway. Our findings suggested that both circ_0001810 and the ERVWE1 protein play significant roles in the pathology of schizophrenia. This research provides new insights into the molecular mechanisms underpinning schizophrenia and may pave the way for novel therapeutic approaches.

## Materials and methods

### Differential expressed genes (DEGs) analysis and gene ontology biological processes (GOBP) analysis

The RNA microarray datasets (GSE53987 [37], GSE25673 [38] and GSE12649 [39]) were obtained from the Gene expression omnibus (GEO) database (http://www.ncbi.nlm.nih.gov/geo/), and based on the GLP570, GPL6244 and GPL96 platform, respectively. DEGs between schizophrenia patients and healthy controls were identified via the GEO2R online tools. GOBP analysis was conducted to evaluate using the Sanger box.

### Clinical samples and ethical statements

This study was carried out following the principles of the Declaration of Helsinki and approved by the Ethics Committee of the School of Basic Medicine of Wuhan University (grant #06R-1366). Blood samples of schizophrenia patients and healthy controls, including the whole peripheral blood (18 schizophrenia patients and 22 healthy controls) for RNA detection, and plasma samples (34 schizophrenia patients and 30 healthy controls) for ELISA analysis, were collected from the Renmin Hospital, Wuhan University. All patients included in the study were recent-onset and met the Diagnostic and Statistical Manual of Mental Disorders, 5th Edition criteria. None of the patients had a history of psychiatric illness. There were no significant differences in median age, education, body mass index (BMI), smoking status, and sex between healthy control and schizophrenia patients. Demographics are presented in Table S1 and S2.

### Plasmid construction

The pCMV-ERVWE1 plasmid was obtained from our lab [13]. The circ_0001810 overexpression vector was constructed by PCR amplifying the full-length exon sequence of circ_0001810, including 1 kb upstream and 1 kb downstream intron sequences at the splice sites. The exon and intron sequences were downloaded from UCSC Genome Browser Home (http://genome.ucsc.edu) and Circbank (http://www.circbank.cn/index.html). Correspondingly, the control vector was designed to delete only the exon sequence of circ_0001810 using the Primer Spanner (http://ps1.biocloud.org.cn). The pCDNA3.1(−) vector was used as the basic vector for the construction of the circ_0001810 overexpression vector and control vector. The full length of circ_0001810 and the 3' untranslated regions (UTRs) of AK2 were amplified into pMIRGlo for the dual-luciferase assay, including the mutative sequences. The AK2 (NM_001625.4) gene was obtained in pCDNA3.1(−) based on the sequence from NCBI. A short hairpin RNA (shRNA) targeting the back-spliced region of circ_0001810 and the control shRNA were cloned into the pSilencer 2.1-U6 neo shRNA expression vector (AM5764, Ambion Inc.). All constructs were confirmed by sequencing (Sangon Biotech, Shanghai). siRNAs against AK2 [40], miR-1197 mimics, biotin-labeled miR-1197 and their NC mimics were obtained from Sangon Biotech (Shanghai, China). Transfection efficiency was measured by RT-qPCR. The sequences and primers are presented in Tables S4 and S5.

### Cell culture and cell transfection

The human neuroblastoma cell line SHSY-5Y (CRL-2266, ATCC) was cultured in a 1:1 mixture of minimum essential medium (MEM) (2225320, Gibco) and F-12 nutrient mixture (2209586, Gibco). The media were supplemented with 10% fetal bovine serum (2001003, Biological Industries), 1% penicillin–streptomycin (2211093, Gibco), and 1% sodium pyruvate (2185865, Gibco). The uninduced SHSY-5Y cells were transfected with the plasmid, and subsequent experiments were conducted 24 h later. Primary neurons in the hippocampus from neonatal *Sprague Dawley* (SD) rats, regardless of gender, were prepared as previously described [41]. Animal experiments were approved by the Animal Ethics Committee of Wuhan University Center for Animal Experiment/A3 Laboratory, Wuhan University. Transfection was performed using Lipofectamine 2000 transfection reagent (11668-019, Invitrogen) according to the manufacturer’s instructions.

### RNA extraction and RNase R treatment

Total RNA was extracted from the whole blood and cells using TRIzol LS reagent (10296028, Invitrogen) and TRIzol reagent (15596018, Invitrogen), respectively. The extracted RNAs were treated with DNase I (18047019, ThermoFisher) to eliminate genomic DNA (gDNA) and then incubated with 3 U/μg RNase R (RNR07250, Epicentre) at 37 °C for 20 min. The treated RNAs were reversely transcribed into cDNA using the PrimeScript™ RT reagent kit (RR037A, Takara) for RT-qPCR after purity and spectrophotometric identification of concentration. Additionally, 1 μg RNA was reverse-transcribed into cDNA using the ReverTra Ace qPCR RT master mix (FSQ-301, Toyobo) for the miRNA detection. GAPDH and U6 were used as controls. All primer sequences are listed in the Table S4 and S5.

### ELISA

The concentration of human AK2 in the plasma of schizophrenia patients and healthy controls was measured using an ELISA kit (E16290h, EIAab biotech) according to the manufacturer’s recommendations. Absorbance was measured at 450 nm using a spectrophotometer. The concentration of AK2 was determined by comparing the optical density of the samples to the standard curve.

### Treatment of actinomycin D (Act D)

To assess the stability of circRNAs, SHSY-5Y cells were treated with 80 μg/mL Act D (HY-17559, MCE) or DMSO (D8371, Solarbio). Cells were collected at different time points (0, 6, 12, 18, and 24 h) to detect the RNA level of circRNA, NCOA2, and GAPDH.

### Subcellular fractionation

SHSY-5Y cells were harvested and resuspended, and the cytoplasmic and nuclear fractions were isolated using the Cytoplasmic and Nuclear RNA Purification Kit (21000, NORGEN), following the manufacturer's instructions. Subsequently, purified RNA was reverse-transcribed for RT-qPCR analysis as previously described. U6 and GAPDH were utilized as internal controls in the nucleus and cytoplasm, respectively.

### RNA immunoprecipitation (RIP)

RIP experiments were conducted referring to the instructions of the Magna RIP RNA-binding protein immunoprecipitation kit (No. 17-700, Millipore). HEK-293T cells were co-transfected with the circ_0001810 overexpression vector and pEnCMV-AGO2 (human)-3 × FLAG (P18498, MiaoLingBio). When the cells reached approximately 90% confluence, they were scraped off and lysed in complete RNA lysis buffer (100 μL of RIP lysis buffer, 0.5 μL of protease inhibitor (ab201119, Abcam), and 0.25 μL of RNase inhibitor (R0102, Beyotime) were added). A 100 μL aliquot of the RIP lysate supernatant was incubated with RIP buffer (860 μL of RIP Wash buffer, 35 μL of 0.5 M EDTA, and 5 μL of RNase inhibitor) containing magnetic beads (20424, Pierce) coated with mouse anti-FLAG antibody (AE005, ABclonal). The negative control group consisted of normal mouse IgG (AC011, ABclonal). After overnight incubation at 4 °C, the co-precipitated RNA was isolated, and its detection was performed using RT-qPCR. The circRNA levels were assayed to determine whether detected circRNA specifically binds to AGO2.

### Dual-luciferase reporter assay

The binding sites between miR-1197 and circ_0001810 or the 3′-UTRs of AK2 were predicted using an online bioinformatics analysis database. The corresponding sequences were amplified and inserted into the pMIRGlo dual-luciferase miRNA target expression vector. Luciferase activity was measured using the Dual-Luciferase Assay System (E1960, Promega) following the manufacturer’s protocol. Firefly luciferase activity was normalized to Renilla luciferase activity.

### RNA pull-down assay

Using the circ_0001810 overexpression vector as a template, we amplified the circ_0001810 mutant fragment (the fragment of circ_0001810 binding to miR-1197) by homologous recombination. The product was then digested with the restriction enzyme *Dpn* I (ER1701, ThermoFisher) and transformed into Escherichia coli to obtain the circ_0001810 mutant vector. Either the circ_0001810 overexpression vector or the circ_0001810 mutant vector was then transfected into SHSY-5Y cells. After 24 h, cells were collected and lysed, with 100 µL of each sample reserved for input analysis. Biotin-labeled miR-1197 (Sangon Biotech, Shanghai) was incubated with streptavidin magnetic beads (P2151, Beyotime), followed by three washes with wash buffer I (10 mmol/L Tris–Cl, 1 mmol/L EDTA, 2 mmol/L NaCl, pH 7.0). The magnetic bead mixture was then combined with the supernatant from cell lysates of either circ_0001810 or circ_0001810-mutant overexpressing SHSY-5Y cells and incubated at 4 °C for 2 h. After washing three times with wash buffer II (50 mmol/L Tris–Cl, 1 mmol/L EDTA, 100 mmol/L KCl, 0.1% Triton-X, 5% glycerin, 1 mmol/L DTT, pH 7.0), RNAs captured were isolated and subjected to qRT-PCR analysis.

### Western blotting

Cells were homogenized and lysed in M-PER™ mammalian protein extraction reagent (UC282138, ThermoFisher) supplemented with protease and phosphatase inhibitors (ab201119, Abcam). Protein concentrations were quantified using the Pierce ™ BCA Protein Assay (UD281372, ThermoFisher). Equal amounts of protein samples were loaded onto 8–12% SDS-PAGE gels, followed by transfer to PVDF membranes (IPVH00010, Amersham Biosciences). The membranes were then blocked with TBST containing 5% nonfat dry milk. The following primary antibodies were used for overnight incubation at 4 °C: OMA1 polyclonal antibody (17116-1-AP, Proteintech), ERVW-1 Rabbit pAb (A16522, ABclonal), DRP1 Rabbit mAb (A21968, ABclonal), MFN1 Rabbit mAb (A21293, ABclonal), MFN2 Rabbit mAb (A19678, ABclonal) and AK2 Rabbit pAb (A6519, ABclonal). GAPDH Mouse mAb (AC002, ABclonal) was used as a control. Subsequently, the membranes were incubated with the secondary antibodies: goat anti-mouse IgG-HRP (AS003, ABclonal), and goat anti-rabbit IgG-HRP (AS014, ABclonal) at room temperature for one hour. The protein bands were visualized using the Millipore Immobilon Western Chemiluminescent HRP Substrate (WBKLS0500, Millipore), and images were captured using the Tanon 5200 chemiluminescence imaging system (Tanon, Shanghai). The expression levels of the target proteins were normalized to GAPDH.

### Determination of cellular ATP levels

Cellular ATP content was determined using the Enhanced ATP Assay Kit (S0027, Beyotime) following the manufacturer’s protocol. Briefly, after 24 h of transfection, SHSY-5Y or primary neuron cells were pretreated and then lysed with ATP lysis buffer. The lysates were centrifuged and 20 μL of each supernatant was quickly mixed with 100 μL of ATP detection working dilution in a 1.5 mL EP tube. Luminescence was immediately measured using a luminometer. The luminescence data were normalized to the protein content of each sample. The data and images were analyzed using GraphPad Prism 5 statistical software.

### Measurement of MMP

Changes in MMP were measured using the JC-1 mitochondrial membrane potential assay kit (C2006, Beyotime) following the manufacturer's instructions. Briefly, the treated cells were harvested and washed twice with PBS. Then, the cells were resuspended in a mixture of 500 μL culture medium and 500 μL JC-1 staining fluid and incubated for 20 min at 37 °C, protected from light. Subsequently, the cells were washed three times with cold staining buffer before analysis using flow cytometry (CytoFLEX S, Beckman Coulter). JC-1 exists either as a cytoplasmic monomer or as mitochondrial J-aggregates, depending on the potential of the mitochondrial membrane. In healthy cells with high MMP, JC-1 spontaneously forms J-aggregates in the mitochondria, emitting red fluorescence. However, in unhealthy cells, the MMP decreases, causing JC-1 to be released from the mitochondria and exist as a monomer in the cytoplasm, resulting in green fluorescence. Thus, the MMP can be indicated by the ratio of red to green fluorescence intensity. Flow cytometry data were analyzed using FlowJo.

### MitoTracker staining and analysis of mitochondrial morphology

Cells cultured on glass coverslips were stained with 100 nM MitoTracker Red CMXRos (M9940, Solarbio) for 25 min at 37 °C, and subsequently counterstained with 4′,6-diamidino-2-phenylindole (DAPI) (RM02978, ABclonal) for 20 min after fixation with 4% paraformaldehyde (PFA) (P0099, Beyotime). The stained cells were imaged using a confocal microscope (Leica-LCS-SP8-STED, Leica Microsystems). At least 50 cells were counted in each sample, and the mitochondrial morphology was categorized as tubular and fragmented, which relatively represented a highly interconnected network of mitochondria, or as small, rod-shaped mitochondria. Cells containing both interconnected and rod-like small mitochondria were classified as intermediate morphotype cells.

### Statistical analysis

All data are presented as the mean ± standard deviation (SD) and were analyzed using GraphPad Prism 8.0.1. Comparisons between two groups were analyzed using *t*-tests or multiple *t* tests, and one-way ANOVA was performed for comparisons between three or more groups. *p* < 0.05 was considered statistically significant.

## Results

### ERVWE1 promoted circ_0001810 production and elevated levels of circ_0001810 positively correlated with ERVWE1 in the blood of individuals

**Fig. 1:**
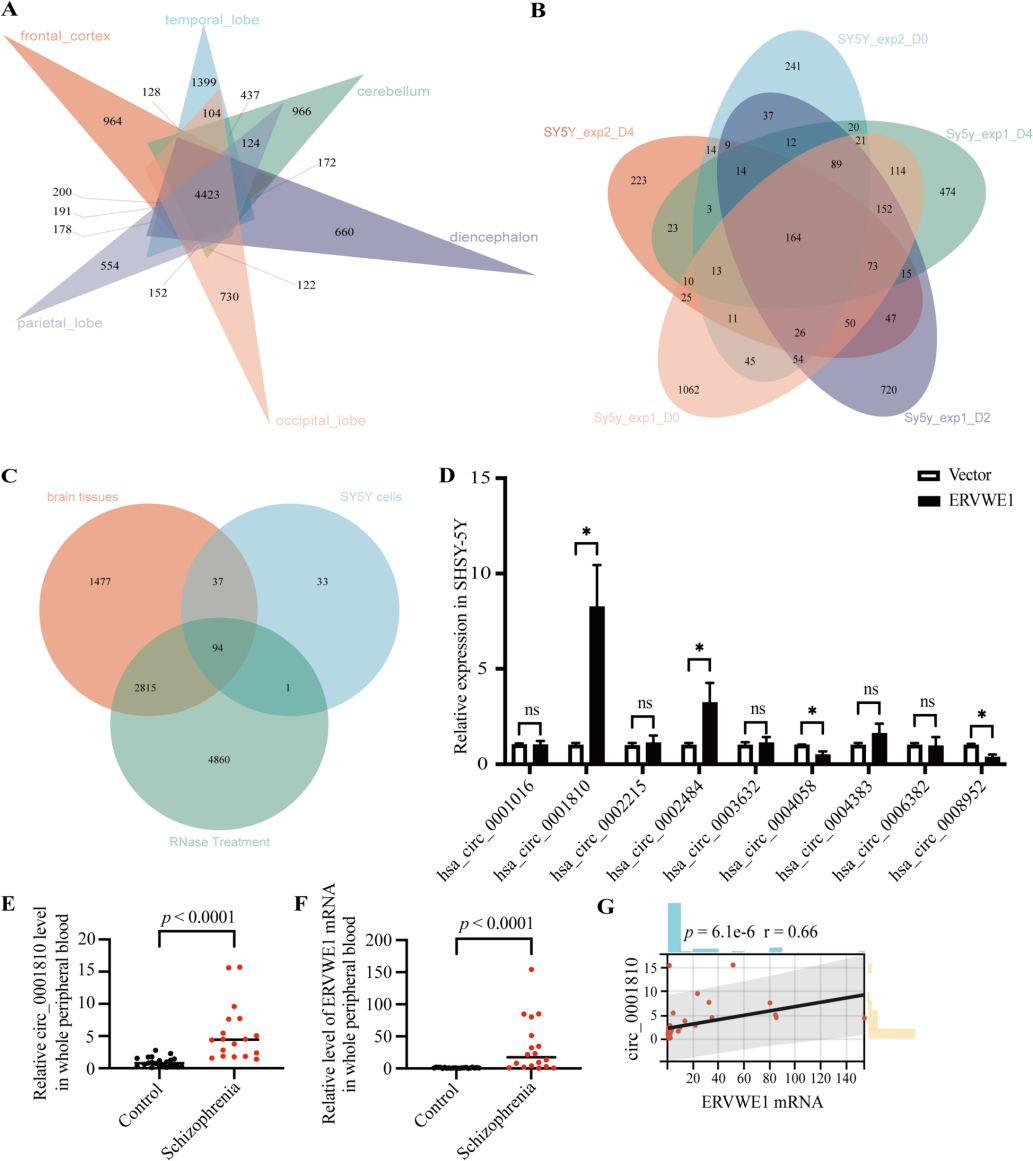
ERVWE1 promoted circ_0001810 production and Elevated Levels of circ_0001810 positively Correlated with ERVWE1. **A** Venn diagram showing the number of circRNAs identified in various brain tissues, as sourced from CircInteractome. **B** Venn diagram showing the number of circRNAs in SHSY-5Y cells from CircInteractome. **C** Venn diagram illustrating the overlap of circRNAs among brain tissues, SHSY-5Y cell lines and the cell treated with RNase. **D** Changes in levels of different circRNAs in SHSY-5Y cells following ERVWE1 overexpression. **D **, **E** Levels of Circ_0001810 in schizophrenia patients (*n* = 18) and the control groups (*n* = 22) measured by RT-qPCR. **F** The mRNA expression of ERVWE1 in schizophrenia patients (*n* = 18) and the control groups (*n* = 22) measured by RT-qPCR. **G** Correlation analysis between the mRNA levels of ERVWE1 and circ_0001810 in schizophrenia patients and healthy controls. The *p*-value of expression by the Mann–Whitney *U* test and the *p*-value in correlation by Spearman. Data were presented as mean ± SD. ^ns^ *p* > 0.05, * *p* < 0.05

The circRNA information from various tissues and cell lines was retrieved from CircInteractome (https://circinteractome.nia.nih.gov). To identify circRNAs potentially involved in schizophrenia, we initially focused on circRNAs present in brain tissues. Research from Max-Delbrück Center for Molecular Medicine in Germany has shown that a total of 65,731 circRNAs are detected in human brain samples, with 21,071 circRNAs in the cerebellum, 24,632 in the diencephalon, 38,983 in the frontal cortex, 31,085 in the occipital lobe, 23,303 in the parietal lobe, and 21,835 in the temporal lobe [42]. Venn diagram analysis showed that 4,423 circRNAs were common across different brain regions, including the frontal cortex, temporal lobe, cerebellum, diencephalon, occipital lobe, and parietal lobe (Fig. 1A). The SHSY-5Y cell line, known for its relevance in neurobiology, served as an effective model for neuronal processes [43, 44]. We selected circRNAs present in SHSY-5Y cells for further screening (Fig. 1B). Additionally, circRNAs are known for their RNase resistance, a characteristic feature [28]. Consequently, we identified 94 circRNAs that met three key screening criteria (Fig. 1C). Based on disease information from circRNADisase v2.0 (http://cgga.org.cn:9091/circRNADisease/) for circRNAs, we focused on circRNAs whose host genes were differentially expressed in SZDB, a schizophrenia database [45]. This process yielded 9 candidate circRNAs (Table S6).

To explore the relationship between ERVWE1 and these circRNAs, we transiently transfected SHSY-5Y cells with an ERVWE1 plasmid and measured circRNA levels (Fig. S1A). This approach allowed us to assess the impact of ERVWE1 on circRNA production specifically. Our findings revealed decreases in hsa_circ_0004058 and hsa_circ_0008952, and increases in hsa_circ_0001810 (circ_0001810) and hsa_circ_0002484 (Fig. 1D). Notably, ERVWE1 overexpression in SHSY-5Y cells significantly increased circ_0001810 production, a result further validated in primary neurons (Fig. S1B-S1C). This indicated a direct regulatory effect of ERVWE1 on circ_0001810 production. Additionally, we found a negative correlation between ERVWE1 and NCOA2 in GSE53987 and GSE25673, which may suggest a potential correlation between circ_0001810 and ERVWE1 in schizophrenia (Fig. S2). Furthermore, we assessed the mRNA expression of NCOA2 and LRCH1, the linear transcripts corresponding to circ_0001810 and circ_0002215, respectively. Results showed that ERVWE1 specifically increased circ_0001810 levels without affecting its linear transcript or broadly altering circRNA levels (Fig. S1D). Thus, circ_0001810 was selected for further study.

We analyzed circ_0001810 expression in schizophrenia patients, marking the first report of its levels in this context. Circ_0001810 was significantly elevated in patient blood samples (*p* < 0.0001, Fig. 1E), with median levels much higher in patients (4.448) than in controls (0.774) (Table 1). Consistent with our previous studies [14, 46], we observed a significant increase in ERVWE1 mRNA in the blood samples of schizophrenia patients (*p* < 0.0001, Fig. 1F), with medians levels of 17.6 in patients compared to 0.919 in controls (Table S3). Next, linear regression analysis showed a positive correlation between circ_0001810 and ERVWE1 (*p* < 0.001, *r* = 0.66, Fig. 1G), with an 80% consistency ratio in mRNA levels between these two markers (Table 2). Univariate and multivariate analyses identified both circ_0001810 and ERVWE1 as potential independent risk factors for schizophrenia (Table 3).

**Table 1:** The RNA level of circ_0001810 in the blood of healthy controls and schizophrenia patients

Control	*n*	22	Schizophrenia	*n*	18
	Mean	0.9379		Mean	5.430
	Median	0.7735		Median	4.448
Standard deviation		0.7421	Standard deviation		4.391
	Skewness	0.9387		Skewness	1.498
	Range	2.669		Range	14.29
	Minimum	0.107		Minimum	1.417
	Maximum	2.776		Maximum	15.70

**Table 2:** The consistency of ERVWE1 mRNA and circ_0001810 expression in schizophrenia patients and healthy controls

		ERVWE1(+)	ERVWE1 (−)	Consistency Ratio
Schizophrenia patients and healthy controls	Circ_0001810 (+)	17	5	80%
	Circ_0001810 (−)	3	15	

ERVWE1 (+): the expression of ERVWE1 above 1.762; ERVWE1 (−): the expression of ERVWE1 below 1.762; Circ_0001810 (+): the expression of circ_0001810 above 1.437; Circ_0001810 (−): the expression of circ_0001810 below 1.437. Clinical data were analyzed by median analysis.

**Table 3:** Univariate and multivariate analysis of risk factors for schizophrenia

Characteristics	Univariate (*p*)	Multivariate			
		OR	95%CI-Lower	95%CI-Upper	*p*
Gender (female vs. male)	0.897				N/A
Age (years)	0.307				N/A
Education (years)	0.061				N/A
BMI	0.734				N/A
Smoking (yes vs. no)	0.565				N/A
ERVWE1	< 0.001	25.827	1.010	660.733	0.049
Circ_0001810	< 0.001	30.583	1.706	548.144	0.020

*N/A* not adopted, *OR* odds ratio, *CI* confidence interval

### Characteristics of circ_0001810 in SHSY-5Y cells: insights into circRNA biology

**Fig. 2:**
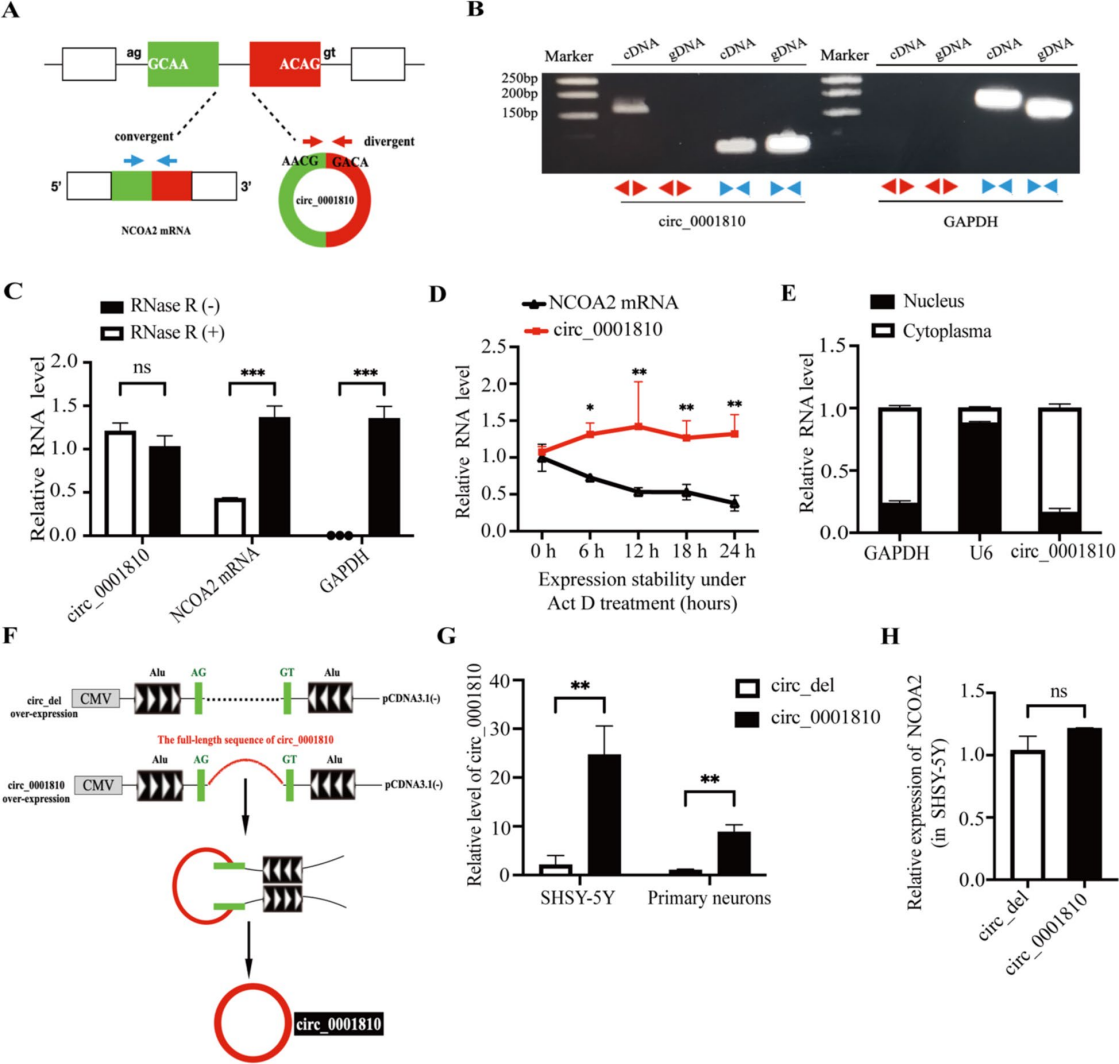
Characteristics of circ_0001810 in SHSY-5Y cells. **A** Design of convergent and divergent primers for circ_0001810 detection. **B** Amplification of circ_0001810 and GAPDH from cDNA and gDNA of SHSY-5Y cells using divergent and convergent primers, respectively. **C** RT-qPCR analysis of circ_0001810, NCOA2, and GAPDH mRNA levels in SHSY-5Y cells with or without RNase R treatment. **D** Stability of circ_0001810 and NCOA2 mRNA in SHSY-5Y cells treated with Act D assessed by RT-qPCR. **E** Subcellular localization of circ_0001810 in SHSY-5Y cells, with GAPDH and U6 as controls. **F** Detailed construction of the circ_0001810 overexpression vector. **G** Detection of circ_0001810 levels following its overexpression in SHSY-5Y cells and primary neurons. **H** The mRNA expression of NCOA2 in SHSY-5Y cells post circ_0001810 overexpression. Each bar represented the mean ± SD of three independent experiments. ^ns^ *p* > 0.05, * *p* < 0.05, ** *p* < 0.01

Given the relatively unexplored nature of circ_0001810, our study aimed to verify that the form expressed in SHSY-5Y cells exhibits typical characteristics of circRNAs. We successfully amplified the full length of circ_0001810 (Fig. S3A). To confirm its circularity, we performed RT-PCR using both convergent and divergent primers (Fig. 2A). The results demonstrated that while convergent primers are standard for linear RNA, the divergent primers uniquely amplified circ_0001810 from cDNA but not from gDNA (Fig. 2B). This pivotal finding establishes the circular nature of circ_0001810. Furthermore, we subjected total RNA to RNase R treatment followed by RT-qPCR analysis. This experiment revealed that circ_0001810 exhibited remarkable resistance to RNase R degradation (Fig. 2C). To further probe its stability, we treated cells with Act D. The resulting data showed that the transcripts of circ_0001810 were considerably more stable than NCOA2 mRNA (Fig. 2D), its linear counterpart. Moreover, subcellular fractionation studies revealed that circ_0001810 predominantly localized in the cytoplasm (Fig. 2E), aligning with the typical distribution pattern of circRNAs. These collective results firmly establish circ_0001810 as a bona fide circRNA.

To investigate the functional role of circ_0001810, we constructed an overexpression plasmid based on the pCDNA3.1(−) vector. Our genomic analysis of circ_0001810 using the UCSC genome Browser identified short interspersed nuclear elements (SINE), specifically from the Alu family, within the intronic regions flanking the exon of circ_0001810 (Fig. S3B). This discovery led us to construct sequences containing Alu elements into the overexpression vector to promote circRNA looping (Fig. 2F). Subsequent experiments utilizing this overexpression vector demonstrated a significant increase in circ_0001810 levels without affecting NCOA2 mRNA levels (Fig. 2G, H). Interestingly, circ_0001810 had no effect on ERVWE1 levels (Fig. S4). This selective upregulation confirms the effectiveness of our vector design and sets the stage for further exploration of circ_0001810's role in cellular processes.

### Circ_0001810 could be a ceRNA of miR-1197

**Fig. 3:**
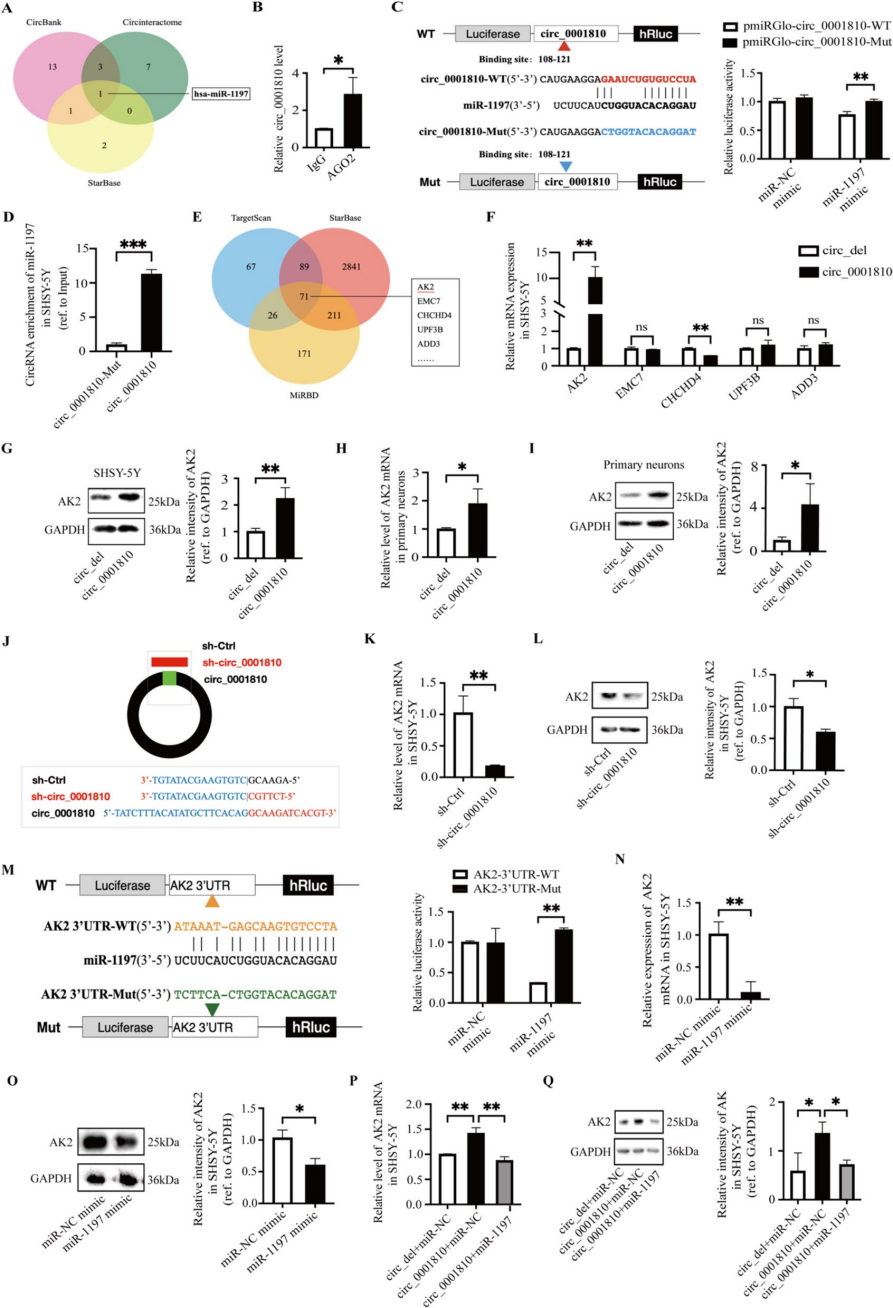
Circ_0001810 acted as a ceRNA of miR-1197, upregulating AK2, the target gene of miR-1197. **A** The miRNA predictions targeting circ_0001810 in StarBase, CircBank, and Circinteractome. **B** RIP assay detected the bind of circ_0001810 to AGO2 protein in HEK-293T cells. **C** Dual luciferase assay examined the bind of circ_0001810 to miR-1197 in SHSY-5Y cells. Mutant circ_0001810 reporter and miR-NC mimic were used as negative controls. **D** RNA pull-down assay examined the in vivo bind of circ_0001810 to miR-1197 in SHSY-5Y cells. Mutant circ_0001810 overexpression vector was used as negative controls. **E** Protein predictions targeting miR-1197 in TargetScan, StarBase, and miRBD. **F** Circ_0001810 upregulated the mRNA expression of different targets in SHSY-5Y cells by RT-qPCR. **G** Circ_0001810 upregulated the protein expression of AK2 by western blots. Results in western blots were quantified using ImageJ and presented as a histogram. **H**, **I** Circ_0001810 upregulated AK2 expression in primary neurons. **J** The plasmid construction of sh-circ_0001810 and its control. **K**, **L** The AK2 expression in SHSY-5Y cells after circ_0001810 knockdown. **M** Dual luciferase assay examined the bind of miR-1197 to the AK2 3′-UTRs in SHSY-5Y cells. The mutant AK2 3′-UTRs reporter and miR-NC mimic were used as negative controls. **N**, **O** The AK2 expression in SHSY-5Y cells post-transfection with miR-1197 or the corresponding control. **P**, **Q** The AK2 expression in SHSY-5Y cells co-transfected with circ_0001810 and miR-1197. 3′-UTRs, 3′-untranslated regions; Mut, mutant; NC, negative control; WT, wild type. Each bar represented the mean ± SD of three independent experiments. ^ns^ *p* > 0.05, * *p* < 0.05, ** *p* < 0.01, *** *p* < 0.001

In our quest to unravel the molecular interactions involving circ_0001810, we utilized three comprehensive databases, including Circbank (http://www.circbank.cn), CircInteractome (https://circinteractome.nia.nih.gov), and StarBase (https://starbase.sysu.edu.cn). These platforms were instrumental in predicting potential miRNAs that might interact with circ_0001810. Among the candidates, miR-1197 emerged as the most likely miRNA to bind to circ_0001810 (Fig. 3A). To further investigate this binding potential, we explored the role of AGO2, a key component in the assembly of miRNAs, known for its involvement in inhibiting the translation of mRNA into proteins [47]. We conducted an RIP experiment to validate the interaction between circ_0001810 and AGO2. The results revealed a significant enrichment of circ_0001810 in micro-ribonucleoproteins containing AGO2 compared to the control IgG (Fig. 3B). This enrichment indicated that circ_0001810 had a potential binding affinity for miRNA. Furthermore, we performed a dual-luciferase reporter assay to confirm this interaction. Intriguingly, the assay demonstrated that the miR-1197 mimic markedly repressed the luciferase activity of the wild-type circ_0001810 reporter, whereas it had no significant effect on the mutant circ_0001810 reporter. Moreover, the luciferase activity of both the wild-type and mutant circ_0001810 reporters remained unchanged in the presence of the miR-NC mimic (Fig. 3C). RNA pull-down assay further confirmed the binding of miR-1197 to circ_0001810. When the binding sequence of circ_0001810 was mutated, the capacity of miR-1197 to interact with it was markedly diminished (Fig. 3D). These findings collectively suggest that circ_0001810 could function as a molecular 'sponge', effectively binding and modulating the activity of miR-1197. This interaction represents a novel aspect of the complex regulatory mechanisms governing gene expression in cellular processes.

### AK2, the target gene of miR-1197, was upregulated by circ_0001810

MiRNAs play crucial roles in cellular processes through the miRNA-mRNA interaction [34]. To identify potential targets for miR-1197, we utilized several databases, including TargetScan (https://www.targetscan.org), StarBase (https://starbase.sysu.edu.cn), and miRBD (http://www.mirdb.org). Our analysis revealed a range of potential targets for miR-1197 (Fig. 3E). The high level of circ_0001810, by sequestering miR-1197, relieved the inhibition of miR-1197 on its targets. Consequently, we examined the effect of circ_0001810 on the mRNA expression of five highly scored targets. The results exhibited that circ_0001810 significantly upregulated AK2 mRNA in SHSY-5Y cells (Fig. 3F) and similarly increased AK2 protein levels (Fig. 3G). These findings were confirmed in primary neurons (Fig. 3H, I). To delve deeper into this relationship, we constructed a knockdown plasmid specifically targeting circ_0001810 (Fig. 3J, S5). Notably, silencing circ_0001810 led to a decrease in AK2 expression (Fig. 3K, L), reinforcing the regulatory link between these molecules. Given our data indicating a tight correlation between circ_0001810 and AK2, we honed in on AK2 as a likely target of miR-1197. To test this hypothesis, we employed a dual-luciferase reporter assay to explore the interaction between miR-1197 and the 3'-UTRs of AK2 (Fig. 3M). Further supporting this interaction, RT-qPCR, and western blot analyses demonstrated that miR-1197 effectively repressed AK2 expression in SHSY-5Y cells (Fig. 3N, O). Moreover, we observed that the upregulation of AK2 expression mediated by circ_0001810 could be attenuated by miR-1197 (Fig. 3P, Q). This finding added another layer to our understanding of the complex regulatory network involving circ_0001810, miR-1197, and AK2. In conclusion, circ_0001810 functioned as a ceRNA for miR-1197, thereby influencing AK2 expression.

### Enrichment of mitochondrial dynamics in schizophrenia dataset, and AK2 induced ATP loss, MMP dissipation and mitochondrial fragmentation

**Fig. 4:**
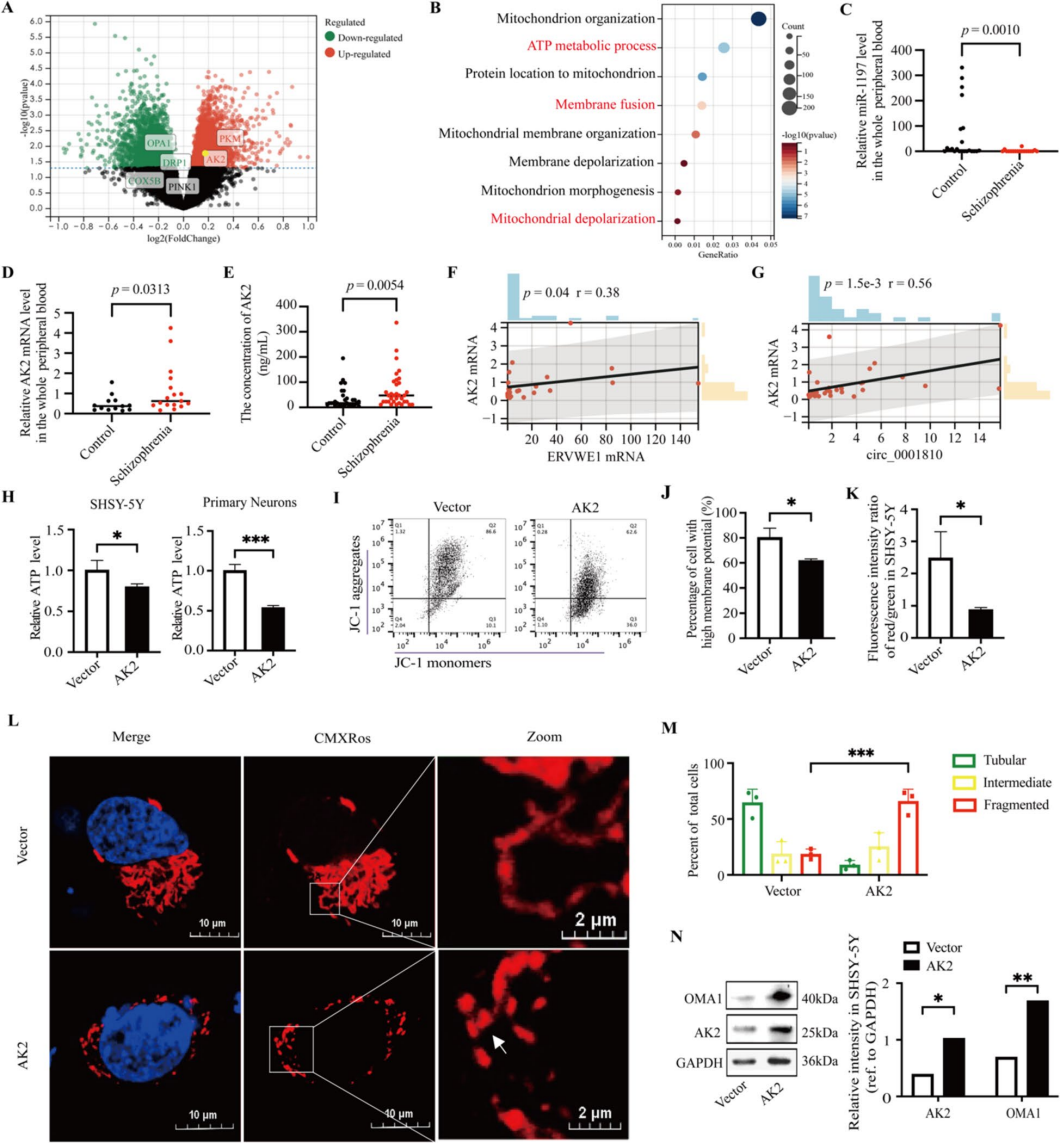
Enrichment of Mitochondrial Dynamics in Schizophrenia Dataset, and AK2 caused ATP loss, MMP dissipation and mitochondrial fragmentation. **A** A volcano plot of the DEGs in the striatum of schizophrenia patients compared to healthy controls in GSE53987. The highlighted dot represented the location of AK2. **B** Partial visualization of GOBP analysis in GSE53987. The X-axis represented gene ratio, and the Y-axis represented different ontologies. The circle color indicated *p*-value and the circle size showed count number. **C** MiR-1197 level in schizophrenia patients (*n* = 18) and the control groups (*n* = 22) measured by RT-qPCR. **D** The mRNA expression of AK2 in schizophrenia patients (*n* = 17) and the control groups (*n* = 13) measured by RT-qPCR. **E** Concentration of AK2 in schizophrenia patients (*n* = 34) and healthy controls (*n* = 30) measured by ELISA. **F**, **G** Correlation analysis of AK2 mRNA with ERVWE1 mRNA, and circ_0001810. The *p*-value of expression by the Mann–Whitney *U* test and the *p*-value in correlation by Spearman. **H** Relative ATP levels in SHSY-5Y cells and primary neurons post AK2 transfection. **I** Flow cytometric dot plots in SHSY-5Y cell induced by AK2. The X-axis represented JC-1 monomers, and the Y-axis represented JC-1 aggregates. Note: AK2-induced MMP dissipation is characterized by the transformation of JC-1 dye aggregates into JC-1 monomers. Each dot represented each cell in a sample. Q2 represented the region where cell with high MMP. **J** Statistical analysis of the percentage of cell with high MMP. **K** The ratios of red/green fluorescence intensity indicative of MMP changes induced by AK2. **L**, **M** Mitochondrial morphology in SHSY-5Y cells, as visualized by MitoTracker Red CMXRos staining post AK2 overexpression. Boxes indicated zoom regions. Arrows represented the sites where mitochondrial fragmentation occurred. Mitochondrial morphology was classified, and the numbers of cells with different morphologies were counted. At least 50 cells per group were counted according to mitochondrial morphologies, and bars represent mean ± SD of three independent experiments. **N** Western blots detecting OMA1 in SHSY-5Y cells after AK2 overexpression. Results were quantified using ImageJ and presented as a histogram. * *p* < 0.05, ** *p* < 0.01, *** *p* < 0.001

The GSE53987 dataset, an RNA microarray dataset from the postmortem striatum of individuals with schizophrenia and healthy controls, revealed elevated AK2 levels (Fig. 4A), hinting at its potential pathophysiological role. Additionally, we observed a similar upregulation of AK2 in the schizophrenia dataset GSE25673 and GSE12649 (Fig. S6). GOBP analysis of GSE53987 indicated that the DEGs, some of which were shown in the volcano plot, were related to the ATP metabolic process, membrane fusion, and mitochondrial depolarization (Fig. 4A, B). Recent studies have consistently implicated mitochondrial dysfunction in the etiology of schizophrenia [48, 49]. Thus, we delved into the association between AK2 and mitochondrial dysfunction in follow-up experiments.

We also measured miR-1197 and AK2 levels in the blood of schizophrenia patients and healthy controls, observing a decrease in miR-1197 levels in schizophrenia patients (*p* = 0.001, Fig. 4C), with medians levels of 0.011 in schizophrenia patients and 4.195 in controls (Table 4). AK2, known to be associated with mitochondrial ATP production [50], was found to be significantly upregulated in the peripheral blood of schizophrenia patients (*p* = 0.0313, Fig. 4D). A comparative study of AK2 protein levels in the plasma of 34 schizophrenia patients and 30 healthy controls revealed a significant increase in the patient group (*p* = 0.0054, Fig. 4E). The median AK2 levels were 47.78 ng/mL in patients, considerably higher than the 17.11 ng/mL in controls (Table 5), indicating a systemic molecular alteration associated with the disorder. Additionally, the correlation analysis indicated a positive association between AK2 mRNA and both ERVWE1 and circ_0001810 in schizophrenia (*p* = 0.04, *r* = 0.38, Fig. 4F;* p* = 0.001, *r* = 0.56, Fig. 4G).

**Table 4:** The RNA level of miR-1197 in the blood of healthy controls and schizophrenia patients

Control	*n*	22	Schizophrenia	*n*	18
	Mean	61.74		Mean	1.94
	Median	4.195		Median	0.011
Standard deviation		107.4	Standard deviation		5.077
	Skewness	1.678		Skewness	3.151
	Range	330.8		Range	20.10
	Minimum	0.01		Minimum	0.01
	Maximum	330.8		Maximum	20.11

**Table 5:** The concentration of AK2 protein in the plasma of healthy controls and schizophrenia patients

Control	*n* (ng/mL)	30	Schizophrenia	*n* (ng/mL)	34
	Mean	34.69		Mean	67.63
	Median	17.11		Median	47.78
Standard deviation		42.00	Standard deviation		71.61
	Skewness	2.41		Skewness	2.15
	Range	190.20		Range	325.20
	Minimum	4.84		Minimum	10.97
	Maximum	195.00		Maximum	336.10

Further exploring the function of AK2, we transiently transfected SHSY-5Y cells and primary neurons with an AK2 overexpression plasmid. Remarkably, AK2 overexpression led to a significant reduction in ATP levels (Fig. 4H), underscoring the complex impact of AK2 on cellular energy metabolism. Flow cytometry analysis revealed a decrease in the percentage of cells with higher membrane potential in Q2 following AK2 overexpression (Fig. 4I, J). Additionally, a decrease in the ratio of red/green fluorescence intensity confirmed MMP decline in SHSY-5Y cells due to AK2 (Fig. 4K). This further underscored the profound effect of AK2 on mitochondrial function. To visualize the impact of AK2 on mitochondrial morphology, we performed an immunofluorescence assay using MitoTracker Red CMXRos in SHSY-5Y cells. The results were striking: AK2 induced substantial mitochondrial fragmentation in approximately 70% of the cells (Fig. 4L, M). This observation suggests a significant disruption of mitochondrial integrity following AK2 upregulation. Given these alterations in ATP production and MMP, we speculated that mitochondrial fragmentation might be due to the activation of OMA1, an MMP-sensitive protease known to influence mitochondrial dynamics. Consistent with our hypothesis, we found that AK2 led to increased OMA1 activation (Fig. 4N), providing vital insight into the mechanistic link between AK2 expression and mitochondrial fragmentation.

### ERVWE1 influenced AK2 through circ_0001810/miR-1197

**Fig. 5:**
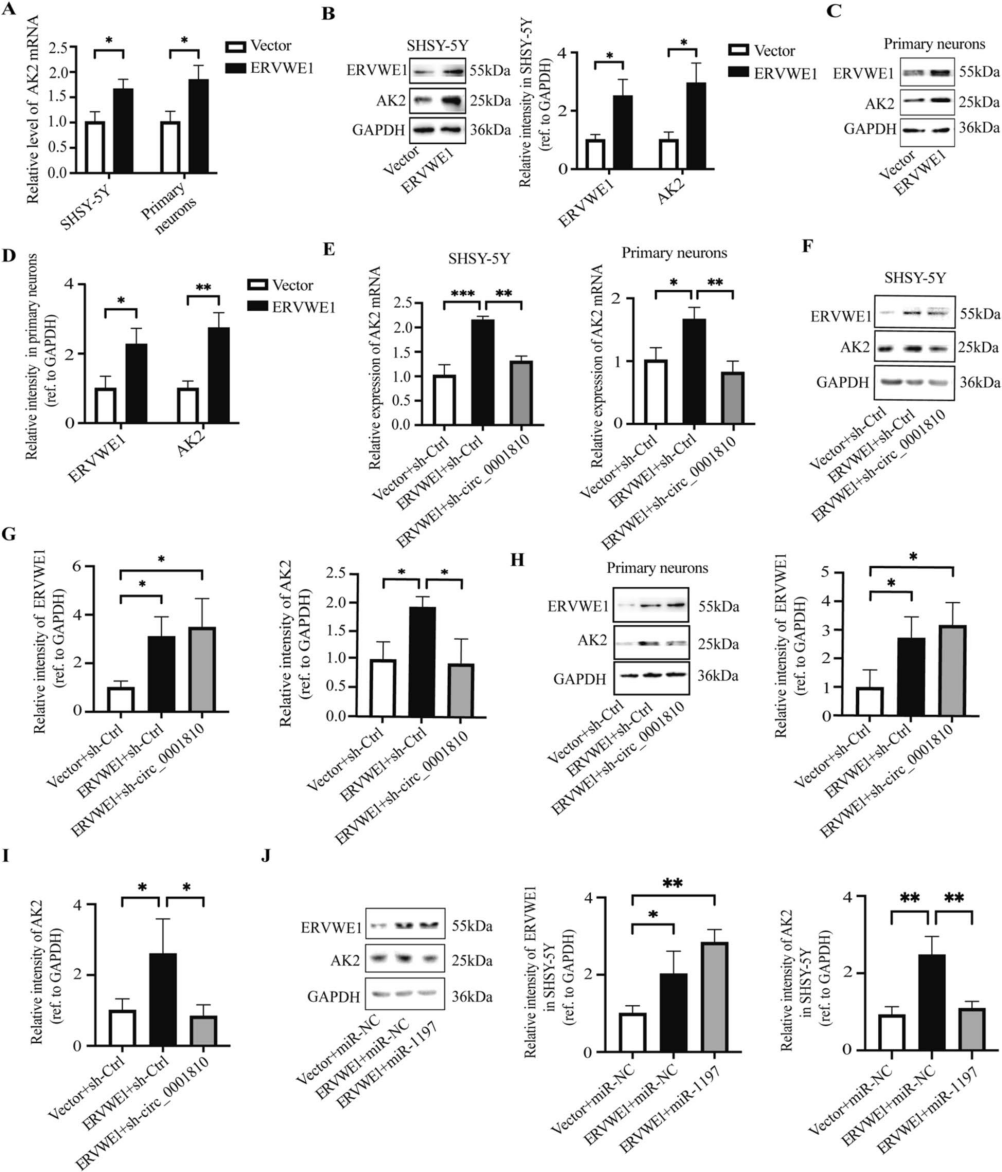
ERVWE1 modulated AK2 function through circ_0001810/miR-1197. **A** RT-qPCR analysis of AK2 mRNA in ERVWE1-expressing SHSY-5Y cells and primary neurons. **B–D** Western blots of AK2 and GAPDH in SHSY-5Y cells and primary neurons transfected with ERVWE1. **E** RT-qPCR analysis of AK2 mRNA levels in SHSY-5Y cells and primary neurons co-transfected with ERVWE1 and sh-circ_0001810. **F–I** Western blots of AK2 and GAPDH in SHSY-5Y cells (**F**, **G**) and primary neurons (**H**, **I**) co-transfected with ERVWE1 and sh-circ_0001810. **J** Western blots of AK2 and GAPDH in SHSY-5Y cells with co-transfected ERVWE1 and miR-1197 mimic. Sh, shRNA. Ctrl, control. Each bar represented the mean ± SD of three independent experiments. * *p* < 0.05, ** *p* < 0.01, *** *p* < 0.001

Our observations from previous experiments painted a complex picture of interactions among ERVWE1, circ_0001810 and AK2. To further unravel this network, we focused on the influence of ERVWE1 on AK2 expression. Our experiments in SHSY-5Y cells and primary neurons provided compelling evidence that ERVWE1 positively regulated AK2 at both the mRNA and protein levels (Fig. 5A–D). Intriguingly, the knockdown of circ_0001810 significantly reversed the ERVWE1-induced increase in AK2 (Fig. 5E–I). This finding underscores the mediating role of circ_0001810 in the ERVWE1/AK2 regulatory axis. Further supporting this, we observed that the enhancement of AK2 levels induced by ERVWE1 could be effectively counteracted by the introduction of a miR-1197 mimic in SHSY-5Y cells (Fig. 5J). These results collectively suggest that ERVWE1 regulates AK2 expression through the circ_0001810/miR-1197 pathway.

In a surprising twist, our study also revealed that ERVWE1 could downregulate the level of miR-1197 (Fig. S7A). Delving deeper, we found that the silencing of circ_0001810 subsequently attenuated the inhibition of miR-1197 by ERVWE1 (Fig. S7B). This discovery adds an additional layer to our understanding of the regulatory dynamics at play, suggesting that ERVWE1 can influence AK2 expression indirectly by modulating miR-1197 levels through circ_0001810.

### ERVWE1 caused ATP loss, mitochondrial depolarization and mitochondrial fragmentation via AK2

**Fig. 6:**
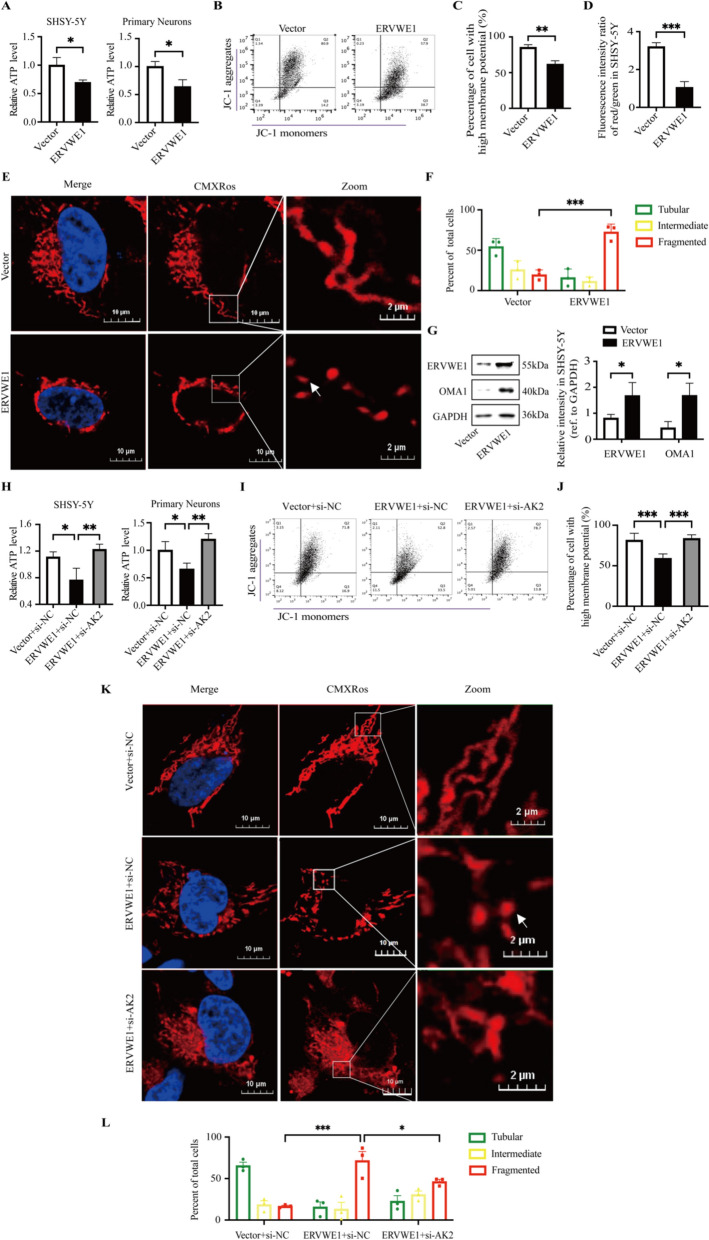
ERVWE1 influenced ATP levels, membrane depolarization and mitochondrial fragmentation via AK2. **A** Relative ATP levels in SHSY-5Y cells and primary neurons post ERVWE1 transfection. **B** Flow cytometric dot plots in SHSY-5Y cell induced by ERVWE1. Note: ERVWE1-induced MMP dissipation is characterized by the transformation of JC-1 dye aggregates into JC-1 monomers. Q2 represented the region where cell with high MMP. **C** Statistical analysis of the percentage of cell with high MMP. **D** The ratios of red/green fluorescence intensity indicative of MMP changes induced by ERVWE1. **E**, **F** Mitochondrial morphology in SHSY-5Y cells visualized by MitoTracker Red CMXRos staining following ERVWE1 transfection. Boxes indicated zoom regions. Arrows represented the sites where mitochondrial fragmentation occurred. At least 50 cells per group were counted according to mitochondrial morphologies. **G** Western blots detecting OMA1 in SHSY-5Y cells post ERVWE1 overexpression. **H** Relative ATP levels in SHSY-5Y cells and primary neurons co-transfected with ERVWE1 and si-AK2. **I**, **J** Flow cytometric dot plots and statistical analysis of the percentage of cell with high MMP induced by co-transfection of ERVWE1 and si-AK2. **K**, **L** Mitochondrial morphology in SHSY-5Y cells visualized by MitoTracker Red CMXRos staining after co-transfection of ERVWE1 with si-AK2. At least 50 cells per group were counted according to mitochondrial morphologies. Bars represented means ± SD of three independent experiments. * *p* < 0.05, ** *p* < 0.01, *** *p* < 0.001

Building upon our understanding of the ERVWE1/circ_0001810/AK2 pathway, we investigated the effect of ERVWE1 on mitochondrial dynamics. The data showed that ERVWE1 led to a significant reduction in ATP levels in both SHSY-5Y cells and primary neurons (Fig. 6A). Furthermore, we observed that ERVWE1 exerted similar effects to those of AK2 overexpression on MMP, leading to mitochondrial depolarization (Fig. 6B–D). Additionally, our results indicated that ERVWE1 not only contributed to mitochondrial fragmentation and but also activated the protease OMA1 (Fig. 6E–G). These findings underscore the significant role of ERVWE1 in altering mitochondrial structure and function. To better characterize the process of mitochondrial fragmentation, we monitored the changes in mitochondrial morphology at 24, 48, and 72 h following ERVWE1 transfection in SHSY-5Y cells. The results showed that mitochondrial fragmentation occurred 24 h post-transfection, and the percentage of cells with fragmented mitochondria increased from 24 to 72 h (Fig. S8). To further validate these observations, we conducted experiments using siRNA specific to AK2 to knock down its expression (Fig. S9). Remarkably, knockdown of AK2 reversed the ERVWE1-induced decrease in ATP levels (Fig. 6H), highlighting the crucial role of AK2 in ERVWE1-mediated cellular energy changes. In line with this, the silencing of AK2 also partially mitigated the decline in MMP induced by ERVWE1 (Fig. 6I, J, S10A), indicating a restorative effect on mitochondrial health. Moreover, the mitochondrial fragmentation observed in SHSY-5Y cells, mediated by ERVWE1, was substantially reduced following AK2 knockdown (Fig. 6K, L). This recovery further confirms the pivotal position of AK2 within the ERVWE1-mediated regulatory pathway affecting mitochondrial integrity.

### Circ_0001810 acted on ERVWE1-induced ATP loss, MMP dissipation and mitochondrial fragmentation

**Fig. 7:**
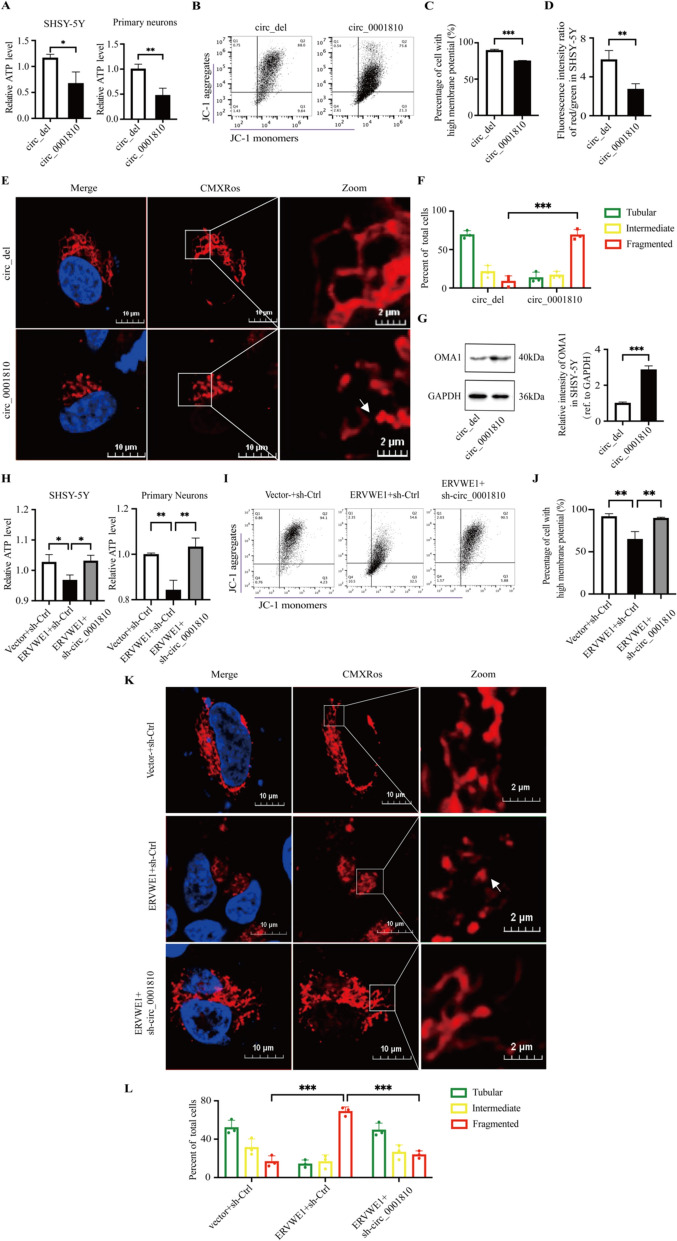
ERVWE1 affected ATP levels, membrane depolarization and mitochondrial fragmentation via circ_0001810. **A** Relative ATP levels in SHSY-5Y cells and primary neurons post circ_0001810 transfection. **B**, **C** Flow cytometric dot plots in SHSY-5Y and statistical analysis of the percentage of cell with high MMP induced by circ_0001810. **D** The ratios of red/green fluorescence intensity indicative of MMP changes induced by circ_0001810. **E**, **F** Mitochondrial morphology in SHSY-5Y cells visualized by MitoTracker Red CMXRos staining post circ_0001810 overexpression. Boxes indicated zoom regions. Arrows represented the sites where mitochondrial fragmentation occurred. At least 50 cells per group were counted according to mitochondrial morphologies. **G** Western blots detecting OMA1 in SHSY-5Y cells following circ_0001810 overexpression. **H** Relative ATP levels in SHSY-5Y cells and primary neurons co-transfected with ERVWE1 and sh-circ_0001810. **I**, **J** Flow cytometric dot plots in SHSY-5Y and statistical analysis of the percentage of cell with high MMP induced by ERVWE1 and sh-circ_0001810. **K**, **L** Mitochondrial morphology in SHSY-5Y cells visualized by MitoTracker Red CMXRos staining following co-transfection of ERVWE1 and sh-circ_0001810. At least 50 cells per group were counted according to mitochondrial morphologies. Each bar represented the mean ± SD of three independent experiments. * *p* < 0.05, ** *p* < 0.01, *** *p* < 0.001

**Fig. 8:**
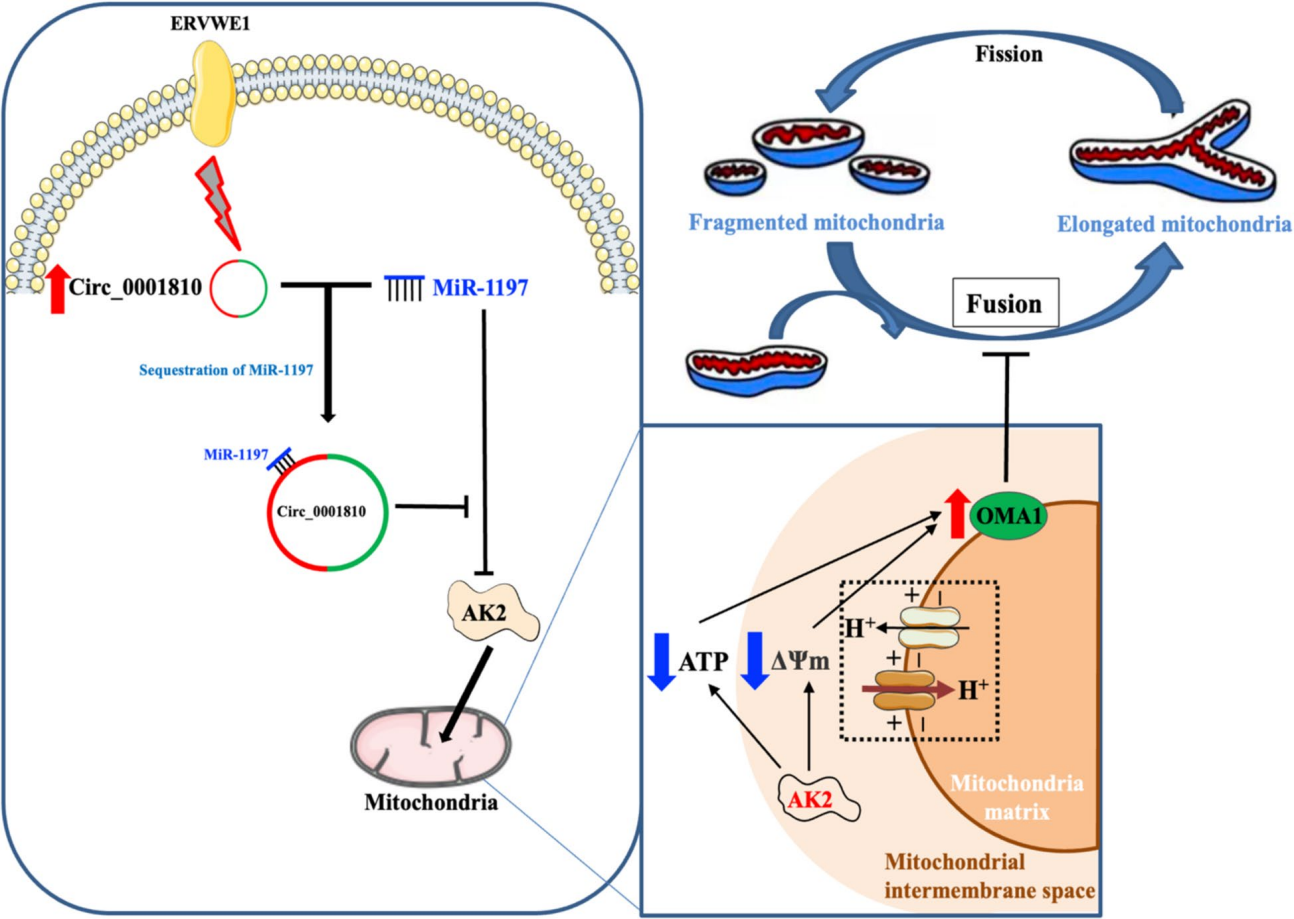
Proposed signaling pathways of ERVWE1 contributing to the mitochondrial dysfunction in neuronal cells via the circ_0001810/miR-1197/AK2 axis

Our experiments focused on the effects of circ_0001810 on mitochondria. We found that circ_0001810 resulted in a significant reduction in ATP level in both SHSY-5Y cells and primary neurons (Fig. 7A). Moreover, circ_0001810 exerted a moderate but noticeable effect on MMP, contributing to a decrease in MMP and promoting mitochondrial fragmentation (Fig. 7B–D). Additionally, our data indicated that circ_0001810 was capable of activating OMA1, a protease involved in mitochondrial dynamics (Fig. 7E–G). In contrast, when we introduced a circ_0001810 knockdown plasmid into cells treated with ERVWE1, ATP loss in SHSY-5Y cells and primary neurons was mitigated (Fig. 7H), demonstrating the regulatory role of circ_0001810 in this process. Interestingly, similar to the effect observed with AK2 silencing, the knockdown of circ_0001810 substantially restored MMP (Fig. 7I, J, S10B) and partially reversed the abnormal mitochondrial morphology induced by ERVWE1 (Fig. 7K, L). This result highlights the interplay between circ_0001810 and AK2 in mediating the effects of ERVWE1 on mitochondrial function. In conclusion, our findings collectively suggested that ERVWE1 impacted ATP levels and regulated mitochondrial dynamic via the circ_0001810/AK2 pathway (Fig. 8). This pathway represents a significant mechanism in cellular energy metabolism and mitochondrial integrity.

## Discussion

The etiology of schizophrenia encompasses both genetic and environmental factors, with genetic contributions considered predominant. Our prior research suggests that ERVWE1 may act as a bridge between genetic and environmental factors, triggering the onset of schizophrenia [13]. For instance, ERVWE1 has been implicated in mediating neuroinflammation by activating the C-reactive protein (CRP) through the TLR3 signal pathway [14], activating cytotoxic T lymphocytes (CTL) [51], enhancing NO production [52], and promoting the expression of IL-10 and TNF-α in glial cells [53]. Additionally, ERVWE1 activates various ion channels, such as small-conductance calcium-activated potassium type 2 channel (SK2) [54], SK3 [55], and transient receptor potential canonical 3 (TRPC3) [56]. It also induces the expression of schizophrenia risk genes [13, 57]. Notably, our lab has discovered that ERVWE1 can lead to organelle abnormalities in neurons, including endoplasmic reticulum stress [58] and damage to mitochondrial energy metabolism [59]. Significantly, ERVWE1 has been observed to disrupt dopaminergic neuron processes [60], impair 5-HT neuronal plasticity [46], and affect hippocampal neuron density and dendritic spine morphology in schizophrenia [61]. Further, our studies suggest that ERVWE1 induces neuronal apoptosis through the lincRNA01930/cGAS/STING pathway [62] and promotes ferroptosis by downregulating GPX4 and SLC3A2 [16]. In this study, we presented clinical data indicating increased levels of circ_0001810 and AK2 in schizophrenia, correlating with ERVWE1. Our extensive investigation demonstrated that ERVWE1 was crucial in reducing intracellular ATP levels, depolarizing mitochondria, and disturbing mitochondrial dynamics via the circ_0001810/miR-1197/AK2 signaling pathway. These findings collectively highlight the significant contribution of ERVWE1 to the etiology of schizophrenia, providing a deeper understanding of its complex biological mechanisms.

In our study, we utilized the SHSY-5Y neuroblastoma cell line and primary SD rat neurons as in vitro models for schizophrenia research. The SHSY-5Y cell line is a thrice cloned subline of SK-N-SH cells, originally established from a bone marrow biopsy of a neuroblastoma patient, with sympathetic adrenergic ganglial origin [63]. Despite this, SHSY-5Y cells are widely used as an in vitro model for schizophrenia research [43, 64, 65]. This is likely due to their neuroblast-like properties, including neuronal marker enzyme activity (tyrosine and dopamine-β-hydroxylases), specific uptake of norepinephrine (NA), and the expression of neurofilament proteins and nerve growth factor receptors [66]. Beyond schizophrenia, SHSY-5Y cells are also frequently used in studies on Parkinson's disease (PD) [67] and Alzheimer's disease (AD) [68]. Moreover, undifferentiated SHSY-5Y cells resemble immature catecholaminergic neurons [69]. A study by scientists from Germany also found that a high number of up-regulated circRNAs already expressed in uninduced SHSY-5Y cells [42]. Therefore, we opted to use uninduced SHSY-5Y cells in the subsequent assay. To enhance the accuracy of brain-related research, primary neurons derived from neonatal SD rats were also used to improve the fidelity of in vivo neuronal simulation [44, 70].

In our research, the degree of ERVWE1 upregulation in neuronal cells following transient transfection mirrored the level of ERVWE1 upregulation observed in the blood of patients with schizophrenia. Furthermore, clinical analysis revealed a positive correlation between circ_0001810 and ERVWE1 in both schizophrenia patients and healthy controls. In vitro study highlighted that ERVWE1 enhanced the expression of circ_0001810 in neuronal cells. In parallel, research in PD has shown that circEPS15 can mitigate neuronal damage [32]. Similarly, in AD, circ-AXL is observed to increase apoptosis rate and reduce neurite outgrowth [71]. These studies collectively underscore the critical role of circRNAs in neurodegenerative diseases. Our study added to this growing body of evidence, suggesting the potential of circRNAs as biomarkers and therapeutic targets in nervous system disorders, including schizophrenia.

Recent studies have revealed that circRNAs are regulated by specific RNA-binding proteins. MBL and QKI have been shown to facilitate back-splicing by bringing splice sites closer together [72, 73]. The double-stranded RNA-editing enzyme ADAR1 and nuclear RNA helicase DHX9 regulate circRNA expression by interacting with double-stranded ALU repeats [74, 75]. A recent study has explored how RBPs regulate circRNAs in the brain. NOVA2, an RBP enriched in neural tissues, regulates circRNA levels by binding to the flanking introns of circRNA loci [76]. Thus, ERVWE1 may affect circRNA biogenesis through its interactions with RBPs in the nervous system. Additionally, our findings that ERVWE1 downregulated other circRNAs suggest that ERVWE1 may not universally increase the levels of circRNAs, offering a nuanced view of its regulatory effects on circRNA expression.

A biomarker is a biological indicator that serves as a surrogate for, and ideally predicts, a clinically relevant endpoint or an intermediate outcome. In clinical settings, the application of clinical biomarkers is often more practical and cost-effective than direct measurements [77]. Schizophrenia, with its complex pathogenesis and diverse symptomatology, presents a significant challenge in early diagnosis. Despite extensive research into schizophrenia, precise biomarkers are scarce. In this context, circRNAs, distinguished by their stable loop structure and the unique head–tail back-spliced site, present distinct advantages. These advantages include extended viability in blood samples and more versatile detection methodologies. Notably, certain circRNAs have been proposed as potential blood biomarkers for mental disorders. For instance, hsa_circRNA_103636 has been proposed as a biomarker for major depressive disorder (MDD) [78]. Similarly, has_circRNA_104597 is among the few circRNAs identified as a potential biomarker for schizophrenia [79]. However, these findings, much like others in the field, are primarily limited to clinical samples and do not delve into the underlying mechanisms. It was important to highlight that our clinical data suggested circ_0001810 as a promising candidate for a novel blood biomarker in the diagnosis of schizophrenia. This discovery opened up new avenues for understanding and potentially diagnosing this complex condition more effectively.

CircRNAs, characterized by their circular structure and lack of free ends, exhibit greater stability compared to linear RNAs, as they are resistant to degradation by exonucleases [80]. This resilience forms the basis for the identification of circRNAs. Specifically, circ_0001810, which we focused on in our study, originates from the NCOA2 gene located on human chr8, encompassing exons 9 and 10 of NCOA2. To confirm the existence of circ_0001810 as a circRNA, we employed several standard assays integral to circRNA research. These included full-length sequencing, which helps in identifying the circRNA integrity, and agarose gel electrophoresis for visualizing the head–tail site of the circular formation. Additionally, we conducted RNase R resistance tests to ascertain the stability of circ_0001810 against exonuclease activity, and RNA stability assays post-Act D treatment, a widely used method to assess RNA degradation rates. Each of these tests, commonly utilized in circRNA studies [81, 82], provided crucial insights into the characteristics and stability of circ_0001810.

Interestingly, many studies have explored the mechanisms of circRNA degradation. First, certain endonucleases, such as RNase H1 and RNase L, participate in circRNA degradation. For instance, ci-ankrd52 is cleaved by RNase H1 when forming R-loops with DNA at its expression sites [83]. Additionally, during viral infection, activated RNase L degrades circRNAs globally. Many circRNAs form 16-26 bp duplexes that act as endogenous inhibitors of PKR, and their degradation by RNase L is crucial for PKR activation [84]. Furthermore, AGO2 plays a role in the degradation of specific circRNAs as a member of the RNA-induced silencing complex (RISC). MiRNA-671 binds extensively to circRNA-Cdr1as, forming the miRNA-671-Cdr1as complex within the nucleus, which is subsequently cleaved and degraded by AGO2 [27, 85]. The m^6^A modification also regulates circRNA degradation [86]. Researchers have identified an endoribonucleolytic cleavage pathway for m^6^A-containing RNAs via the YTHDF2-HRSP12-RNase-P/MRP pathway, where HRSP12 serves as an adapter between YTHDF2 and RNase-P/MRP to promote the rapid degradation of YTHDF2-bound m^6^A-containing circRNAs [87, 88]. Lastly, structure-mediated RNA decay (SRD) governs the degradation of highly structured RNA molecules, characterized by a highly folded structure in their 3' UTRs. This process involves the RNA-binding proteins UPF1 and G3BP1, which bind to highly structured base-pair regions in circRNAs, directing their degradation [89].

The functional roles of circRNAs are often linked to their cellular localization [28]. For instance, circRNAs, localized primarily in the nucleus, can interact with U1 small nuclear ribonucleoprotein (snRNP), enhancing the transcription of their parental genes [90]. In contrast, the abundance of circRNAs in the cytoplasm, coupled with their numerous miRNA binding sites, highlights their capacity to regulate cellular functions by acting as miRNA sponges. This is exemplified by circRNAs like CDR1as [91]. In this study, we observed a significant cytoplasmic localization of circ_0001810. Considering predictions from various database, it seemed plausible that circ_0001810 primarily functions by sequestering miR-1197. The AGO2, a central component of RISCs that facilitates RNA interference and gene silencing, is found to bind to circ_0001810. This interaction suggests a potential mechanism of circRNA-miRNA crosstalk, as demonstrated in RIP assays. Further confirmation came from dual-luciferase reporter assays, which verified the binding ability of miR-1197 to circ_0001810, suggesting its role as a novel miR-1197 sponge. Similar binding to miR-1197 have also identified for circRNA_0000429, circRNA_0075542, circRNA_0004018, and circRNA_0049271. Researches indicate that circ_0000429 and circ_0049271 are upregulated in non-small cell lung cancer (NSCLC) and acute myocardial infarction (AMI), respectively [92, 93]. Conversely, circ_0075542 and circ_0004018 are downregulated in human prostate tumor tissues and hepatocellular carcinoma (HCC), respectively [38, 94]. Additionally, as a molecular sponge, circ_0001810 may also influence the transcription factors of miR-1197. For instance, the miR-132/212 and miR-379–410 clusters are regulated by neuronal activity-induced transcription factors like CREB and MEF2 [95, 96]. Therefore, we speculated that the production of miR-1197 might be triggered in a manner akin to the activity-induced activation of protein-coding gene transcription, offering new insights into the complex regulatory networks within neuronal cells.

MiRNAs are known to modulate protein functions by expediting mRNA degradation and reducing mRNA translation. It has been discovered that miR-1197 binds to various proteins, notably PTEN [97] and PPARGC1A [98]. These proteins are implicated in development of autism spectrum disorders (ASDs) and PD, respectively. In this study, we observed that miR-1197 inhibited AK2 in neuronal cells. AK2 is a protein whose levels are elevated in the rat hippocampus, playing an essential role in temporal lobe epilepsy (TLE) [99]. We found that circ_0001810 upregulated AK2 through miR-1197 in neuronal cells. Similar molecular mechanisms of circRNA/miRNA/mRNA have also been elucidated in PD [100] and AD [71]. Additionally, studies have demonstrated that certain microbial protein, such as the parasites' transferase [101], influence AK2 protein. Our findings suggested that ERVWE1 impacted AK2 through circ_0001810/miR-1197 in neurons. This discovery adds to the growing body of evidence about the intricate molecular interactions in neurological conditions, offering new insights into the potential therapeutic targets for these disorders.

AK2 plays a crucial role in regulating the synthesis and consumption balance of the adenine nucleotide pool, which suggested that AK2 may affect ATP level [102]. In this study, we noted a decrease in ATP levels associated with AK2. This observation aligns with findings in astrocytes, where gene expression changes involved in ATP regulation, and resulting abnormalities in adenosine metabolism, have been proposed as a potential pathophysiological mechanism in schizophrenia [103]. Other research underscores the impact of certain circRNAs on ATP level. For instant, circPUM1, found in mitochondria, is known to boost oxidative phosphorylation, thereby enhancing ATP production in the context of pyroptosis [104]. In our research, we observed that circ_0001810 led to a reduction in ATP level. This finding is paralleled in studies of certain viral proteins, such as the HIV-1 viral protein R, which are also known to decrease ATP levels [105]. Crucially, we revealed that ERVWE1 reduced ATP level in neuronal cells through AK2. This alteration in ATP has been previously reported in the frontal and left temporal lobes of individuals with schizophrenia [106]. These findings suggest a possible mechanism where ERVWE1, through the circ_0001810/miR-1197 pathway, might contribute to reduced ATP levels in schizophrenia. This insight offers a new perspective on the metabolic alterations occurring in this complex psychiatric disorder.

AK2, intriguingly, demonstrates a range of effects that extend beyond its known physiological functions in mitochondria. Significantly, during apoptosis, AK2 plays a role in mitochondrial polarization [107]. Assessing mitochondrial function critically involves measuring the MMP, which accounts for over 90% of the total available respiratory energy [108]. Mitochondrial depolarization, for instance, has been detected in the brain's isolated mitochondria in rat models of schizophrenia [109]. In our data, we observed that AK2 instigated mitochondrial depolarization, implying that AK2 might be a key player in the pathogenesis of schizophrenia. This finding aligns with reports that circ_002664 mediates MMP dissipation [110]. In our research involving circ_0001810, we noted a similar impact on MMP. A previous study from our lab establishes that ERVWE1 reduces MMP, contributing to neuronal ferroptosis [34]. Building on this, our current study revealed that ERVWE1 induced mitochondrial depolarization in neurons through circ_0001810/miR-1197/AK2 pathway. This novel insight into the ERVWE1's role in mitochondrial dynamics furthers our understanding of its potential impact in the pathophysiology of schizophrenia.

Mitochondrial depolarization is known to trigger the activation of proteins that play a role in mitochondrial dynamics. Under normal physiological conditions, mitochondrial fission and fusion are crucial for maintaining mitochondrial function by facilitating the clearance of damaged mitochondria and ATP synthesis, respectively [23]. However, these processes are tightly regulated in response to various stimuli. For instance, during starvation and stress conditions, fission is inhibited, and fusion is induced, resulting in a highly interconnected mitochondrial network [111–113]. Conversely, severe mitochondrial damage and depolarization impair fusion, leading to unopposed fission and mitochondrial fragmentation [114, 115]. Depolarization-induced fragmentation has been proposed to occur due to the degradation of key components in the mitochondrial fusion machinery, specifically OMA1 peptidase, which mediates the degradation of long isoforms of dynamin-like GTPase OPA1 in the inner membrane. This ultimately inhibits fusion and triggers mitochondrial fragmentation [116, 117]. In our study, we observed that fusion was inhibited following depolarization-induced mitochondrial changes, which subsequently led to mitochondrial fragmentation. OMA1 activation by AK2 accelerated mitochondrial fragmentation. Furthermore, aggravated fission or inhibited fusion results in mitochondrial fragmentation. We also found that ERVWE1 increased DRP1 expression independent of circ_0001810, suggesting ERVWE1's effect on the fission process (Fig. S11), which warrants further exploration. Mitochondrial fragmentation has been observed in the post-mortem prefrontal cortex of schizophrenia patients [118]. Further supporting our findings, studies have demonstrated that circIgfbp2 regulates mitochondrial dysfunction in the brain [33]. We discovered that circ_0001810 also played a role in the activation of OMA1 and mitochondrial fragmentation. This is corroborated by research in liver injury, where circ-CBFB was found to upregulate OMA1, and disturb mitochondrial dynamics [119]. Moreover, viral infections like Hepatitis C virus (HCV) infection and influenza A virus (IAV) have been shown to induce mitochondrial fragmentation and mitophagy, respectively [120]. Moreover, a circRNA protein induced by transmissible gastroenteritis virus (TGEV), encoded by circBIRC6-2, contributes to mitochondrial dysfunction [121]. Our data suggested that ERVWE1 accelerated OMA1 activation and mitochondrial fragmentation. These align with clinical studies that have linked mitochondrial dysfunction with an increased risk of developing schizophrenia [122].

An impaired cellular energy state, accompanied by dysfunctional mitochondria, is a strong hypothesis for the vast heterogeneity observed in schizophrenia [123]. Interestingly, the findings by Yao et al. found that inhibited fusion causes the slight increase in ATP levels in cell proliferation [124], which might be the difference between cancer cells and neural cells. Researches has shown that neuronal development varies between species, with the slow development of the human brain contributing to its complexity. The pace of neuronal development is closely linked to mitochondrial metabolic activity [125]. Hence, we hypothesize that nerve cells lack the rapid energy compensation seen in cancer cells. Our findings proposed that ERVWE1 influenced mitochondrial dysfunction through circ_0001810/miR-1197/AK2, which could play a significant role in the pathology of schizophrenia. Furthermore, several studies also suggest that aberrant mitochondrial dynamics may contribute to abnormal brain connectivity in individuals with schizophrenia [22, 126]. This insight underscores the importance of understanding mitochondrial dynamics in the broader context of schizophrenia pathology.

The development of drugs for schizophrenia has traditionally centered on molecular targets like dopamine, serotonin, and adrenaline receptors. However, schizophrenia characterized by three groups of symptoms, and current antipsychotics often show greater efficacy in treating positive symptoms compared to negative symptoms or cognitive deficits. Moreover, some antipsychotics are linked to severe neurological and metabolic side effects, including sexual dysfunction and agranulocytosis, as seen with drugs like clozapine. Consequently, there has been a shift towards developing disease-specific that target the core pathology of schizophrenia, addressing both positive and negative symptom domains [127]. In this context, ERVWE1 has emerged as a promising treatment target for schizophrenia treatment due to its essential role in the pathophysiology of the disorder. Notably, GNbAC1, a humanized IgG4 monoclonal antibody that specifically targets ERVWE1, has already been used in a year-long phase 2b clinical trial for multiple sclerosis [128]. Its potential is further highlighted by its prospects in clinical trials for type 1 diabetes (T1D) [129]. These developments suggest that a monoclonal antibody targeting ERVWE1 could offer significant promise as an innovative therapeutic approach for schizophrenia. This approach reflects a new direction in the ongoing quest to improve treatments for this complex mental health condition.

## Conclusions

In our research, we uncovered a novel pathway, ERVWE1/circ_0001810/miR-1197/AK2 that significantly influenced mitochondrial energy and morphology in schizophrenia. This groundbreaking discovery opens up new avenues for diagnosing and treating schizophrenia, positioning circ_0001818 as a potential biomarker for the condition. Although circRNAs have not yet been tested in clinical trials, recent advancements in nucleotide drug delivery technology are promising. For instance, the rabies virus glycoprotein-circSCMH1-extracellular vesicles have shown effectiveness in rodent and nonhuman primate ischemic stroke models of ischemic stroke [130]. This breakthrough provides solid technical foundation for the potential application of circRNA in clinical settings. We are optimistic that further research and clinical data will deepen our understanding of circ_0001810's role in schizophrenia treatment. This area of study represents a promising new frontier in the ongoing quest to develop more effective strategies for managing this complex mental health condition.

## Supplementary Information

Additional file 1.

Additional file 2.

Additional file 3.

## Data Availability

All data is available from the corresponding author upon request.

## Abbreviations

Act D: Actinomycin D
AD: Alzheimer's disease
AK2: Adenylate kinase 2
ASDs: Autism spectrum disorders
BMI: Body mass index
ceRNA: Competing endogenous RNA
CircRNAs: Circular RNAs
CRP: C-reactive protein
CTL: Cytotoxic T lymphocytes
DAPI: 4',6-Diamidino-2-phenylindole
DEGs: Differential expressed genes
gDNA: Genomic DNA
GEO: Gene expression omnibus
HCV: Hepatitis C virus
HERVs: Human endogenous retroviruses
IAV: Influenza A virus
MDD: Major depressive disorder
MMP: Mitochondrial membrane potential
PD: Parkinson's disease
PFA: Paraformaldehyde
RIP: RNA immunoprecipitation
RISCs: RNA-induced silencing complexes
shRNA: Short hairpin RNA
SD: Sprague Dawley
SINE: Short interspersed nuclear elements
SK: Small-conductance calcium-activated potassium type
snRNP: Small nuclear ribonucleoprotein
T1D: Type 1 diabetes
TGEV: Transmissible gastroenteritis virus
TLE: Temporal lobe epilepsy
TRPC3: Transient receptor potential canonical 3
UTRs: Untranslated regions
