# Supplementary material for: Ancestral retrovirus envelope protein ERVWE1 upregulates circ_0001810, a potential biomarker for schizophrenia, and induces neuronal mitochondrial dysfunction via activating AK2
Source: Cell Biosci. 2024 Nov 14;14:138. doi: 10.1186/s13578-024-01318-1 (PMC11566632; doi:10.1186/s13578-024-01318-1)
Supplement: Supplementary file 1 — Additional file 1. [file 13578_2024_1318_MOESM1_ESM.docx]

**SUPPLEMENTARY MATERIAL 1**

**Ancestral Retrovirus Envelope Protein ERVWE1 Upregulates Circ_0001810, a Potential Biomarker for Schizophrenia, and induces Neuronal Mitochondrial Dysfunction via activating AK2**

Running title：ERVWE1 induces Mitochondrial Dysfunction via circ_0001810/AK2 pathway in schizophrenia

Wenshi Li, Xing Xue, Xuhang Li, Xiulin Wu, Ping Zhou, Yaru Xia, Jiahang Zhang, Mengqi Zhang, Fan Zhu#

State Key Laboratory of Virology, Department of Medical Microbiology, School of Basic Medical Science, Wuhan University, Wuhan, 430071, China

#Address correspondence to Fan Zhu, Professor, Wuhan 430072, P. R. China, Tel: 86-027-68759906, Fax: 86-027-68759906, E-mail: [fanzhu@whu.edu.cn](mailto:fanzhu@whu.edu.cn). ORCID: 0000-0001-7031-2956.

**Supplementary Tables**

**Table S1** Comparison of the whole peripheral blood samples demographic data between the healthy controls and schizophrenia patients

|  | Schizophrenia (*n*=18) | | Controls (*n*=22) | | Analysis |
| --- | --- | --- | --- | --- | --- |
|  | Median | Range | Median | Range | *p* |
| Age (year)^a^ | 46 | 29-66 | 38 | 23-65 | 0.3146 |
| Education (year)^a^ | 12 | 3-12 | 12 | 6-16 | 0.0541 |
| BMI (body mass index) ^a^ | 20.3 | 16.9-26.4 | 21.9 | 15.6-26.4 | 0.8402 |
|  | N | % | N | % | *p* |
| Gender^b^ |  |  |  |  |  |
| Male | 7 | 39 | 9 | 41 | 0.8968 |
| Femal | 11 | 61 | 13 | 59 |  |
| Smoking status^b^ |  |  |  |  |  |
| Yes | 5 | 27 | 8 | 39 | 0.5641 |
| No | 13 | 73 | 14 | 61 |  |

Notes: a-*p* values were calculated by the Mann-Whitney U test.

b-*p* values were calculated by the chi-square test.

**Table S2** Comparison of the plasma samples demographic data between the healthy controls and schizophrenia patients

|  | Schizophrenia (*n*=34) | | Controls (*n*=30) | | Analysis |
| --- | --- | --- | --- | --- | --- |
|  | Median | Range | Median | Range | *p* |
| Age (year)^a^ | 38 | 20-66 | 46 | 23-64 | 0.1617 |
| Education (year)^a^ | 12 | 3-16 | 12 | 3-16 | 0.2662 |
| BMI (body mass index) ^a^ | 22.5 | 16.9-26.4 | 22.1 | 15.1-27.4 | 0.9388 |
|  | N | % | N | % | *p* |
| Gender^b^ |  |  |  |  |  |
| Male | 16 | 47 | 13 | 43 | 0.7651 |
| Femal | 18 | 53 | 17 | 57 |  |
| Smoking status^b^ |  |  |  |  |  |
| Yes | 14 | 41 | 10 | 33 | 0.5178 |
| No | 20 | 59 | 20 | 67 |  |

Notes: a-*p* values were calculated by the Mann-Whitney U test.

b-*p* values were calculated by the chi-square test.

**Table S3** The mRNA level of ERVWE1 in the blood of healthy controls and schizophrenia patients

| **Control** | ***n*** | **22** | **Schizophrenia** | ***n*** | **18** |
| --- | --- | --- | --- | --- | --- |
|  | mean | 1.070 |  | mean | 33.99 |
|  | median | 0.919 |  | median | 17.60 |
| Standard deviation | | 0.619 | Standard deviation | | 42.17 |
|  | Skewness | 0.429 |  | Skewness | 1.657 |
|  | Range | 1.899 |  | Range | 153.8 |
|  | Minimum | 0.189 |  | Minimum | 0.654 |
|  | Maximum | 2.088 |  | Maximum | 154.4 |

**Table S4** The sequences for miRNA and siRNA

| Gene | Sense (5’-3’) | Antisense (5’-3’) |
| --- | --- | --- |
| MiR-1197 mimic | UAGGACACAUGGUCUACUUCU | AGAAGUAGACCAUGUGUCCUA |
| MiR-NC mimic | UUUGUACUACACAAAAGUACUG | CAGUACUUUUGUGUAGUACAAA |
| Biotin-miR-1197 | UAGGACACAUGGUCUACUUCU | AGAAGUAGACCAUGUGUCCUA |
| Biotin-miR-NC | UUUGUACUACACAAAAGUACUG | CAGUACUUUUGUGUAGUACAAA |
| si-AK2 | GCCACATGTAAAGACTTGGTT | AACCAAGUCUUUACAUGUGGC |
| si-NC | UUCUCCGAACGUGUCACGUTT | ACGUGACACGUUCGGAGAATT |

**Table S5** The primers in PCR

|  | Forward Sequence | Reverse Sequence |
| --- | --- | --- |
| Circ_0001016  for RT-qPCR | AAGATTCTTCCAAGGAACCAGTG | GACTCTTGTCCAAGCATCAGGAT |
| Circ_0002215  for RT-qPCR | TCACCAGCGGTTCCCAAAC | CTCACGAAGTTGTTCCACC |
| Circ_0002484  for RT-qPCR | CCAGCCTGTCAGTGCATCT | CTGAGGACACGGGAGAAAT |
| Circ_0003632  for RT-qPCR | GTTGTATGGCTCATCTCTGACTAGA | TTTCAATGTGAATTAAGCAAAGTTT |
| Circ_0004058  for RT-qPCR | AGAGACTGGGGTGTGGAAAAA | CAGAGGTAAGATAAGGTCGGG |
| Circ_0004383  for RT-qPCR | CCAGGAACCATTGGATAAGGA | GAGGTAAGATAAGGTCGGGCT |
| Circ_0006382  for RT-qPCR | GCTGCGGAAGTCCTAAACAAG | CGTCCATTAAGAGCTCCACGT |
| Circ_0008952  for RT-qPCR | TTACACTCAACTAATTGGCATCA | CATGTCTAAAAACAAAGAGGTCC |
| Circ_0001810 for RT-qPCR | TGCTTCACAGGCAAGATCACG | ACCTTCTTACCAGGTCCTCCC |
| Circ_0001810 for overexpression step 1 | CCGCTCGAG TTATTAACGTTTTAGTGTAG | CGCGGATCC AAAATATTATTTGTAAATATAT |
| Circ_0001810 for overexpression step 2 | TAAGAGGCATCATCATGAAGTACTGAGACAAGGATTGGCA | TGCCAATCCTTGTCTCAGTACTTCATGATGATGCCTCTTA |
| Circ_del for circRNA control | CTTAATAGGTAATCCTAGTTCTAAGAAGTCAT | AGGATTACCTATTAAGTAACAGCAGGCATTATAC |
| sh-Circ_0001810  for knockdown | GATCCTCTTGCCTGTGAAGCATATGTTTCAAGAGAACATATGCTTCACAGGCAAGATTTTTTGTCGACA | AGCTTGTCGACAAAAAATCTTGCCTGTGAAGCATATGTTCTCTTGAAACATATGCTTCACAGGCAAGAG |
| sh-Ctrl | GATCCAGAACGCTGTGAAGCATATGTTTCAAGAGAACATATGCTTCACAGCGTTCTTTTTTTGTCGACA | AGCTTGTCGACAAAAAAAGAACGCTGTGAAGCATATGTTCTCTTGAAACATATGCTTCACAGCGTTCTG |
| Circ_0001810  for divergent primer | CCTTGTCTGATGGCACTCTTG | ACCTTCTTACCAGGTCCTCCC |
| Circ_0001810  for convergent primer | CCATGAGAGCAGCCATGAAAC | TGCGCATGGAACTTCTGAATAC |
| MiR-1197 for reverse transcription | GTCGTATCCAGTGCAGGGTCCGAGGTATTCGCACTGGATACGACAGAAGTA |  |
| MiR-1197 for  RT-qPCR | GCTAGGACACATGGTCTACTTCT | GCAGGGTCCGAGGTATTC |
| pMIGlo-circ_0001810wt | CTAGCTAGCGCAAGATCACGTCTCTGGAT | TGCTCTAGACTGTGAAGCATATGTAAAG |
| pMIGlo-circ_0001810mut | CTGGTACACAGGATTGCTAAGAGGCATCATCATG | CCTGTGTACCAGTCCTTCATGCTGCGCATG |
| ERVWE1 | CCATGCCGCTGTATGACCAG | GGGTTCCCTTAGAAAGACTCCT |
| NCOA2 | GCTGGGGCCTATGATGCTTG | GACAAATTCCGTGTGGTCCC |
| LRCH1 | ACTGTTGACACTCTGCTGGC | CAGAGCACAGCGTTTGGATG |
| U6 | CTCGCTTCGGCAGCACA | AACGCTTCACGAATTTGCGT |
| AK2 for RT-qPCR | GGTAAAGGGACCCAGGCAC | CACCAGTTTCCCAGCATCCA |
| EMC7 for RT-qPCR | ACGGGAAATGGAGCAGTCAA | ACTACCTCCTTTTGCCAGCC |
| CHCHD4 for RT-qPCR | ACGCGTCCGAGAGGAGAG | TCAGCCACCAATTCTGCACT |
| UPF3B for RT-qPCR | AGCAATCCAGAGGTGCCTTC | TGTCCTTGCCGTCACCTTTT |
| ADD3 for RT-qPCR | TGTGACTGCTTTTTCCTTTG | GGTGAGTTCAGCCATCTTGT |
| AK2  for overexpression | CTAGCTAGCGCCACCATGGCTCCCAGCGTGCCAG | CGGGGTACCTTAGATAAACATAACCAAG |
| pMIGlo-AK2-wt-3UTR | CTAGCTAGCTCAACAAATCAAGCAAAC | GCTCTAGATTCTAGCTTAGATTATCT |
| pMIGlo-AK2-mut-3UTR | TCTTCACTGGTACACAGGAT AGCTATGTCATCCAAAGAT | CCTGTGTACCAGTGAAGATTAAAAGAGTCCCTTCACAC |
| GAPDH for PCR | ATGACATCAAGAAGGTGGTG | CATACCAGGAAATGAGCTTG |
| GAPDH  for divergent primer | TTGCCCTCAACGACCACTTT | GACCAAATCCGTTGACTCCG |
| DRP1 for RT-qPCR | CTGGTCCTCGTCCTGCTTTAT | TGATCCTTTGCATTTCCTCAT |
| MFN1 for RT-qPCR | CTTGAGAGATGACCTGGTGTT | AGTGTTGATTCAGAGTTTGCG |
| MFN2 for RT-qPCR | TGTTGTTGGAGGAGTGGTGTG | CAGTTGGAGCCAGTGTAGCTG |

**Supplementary Figures**


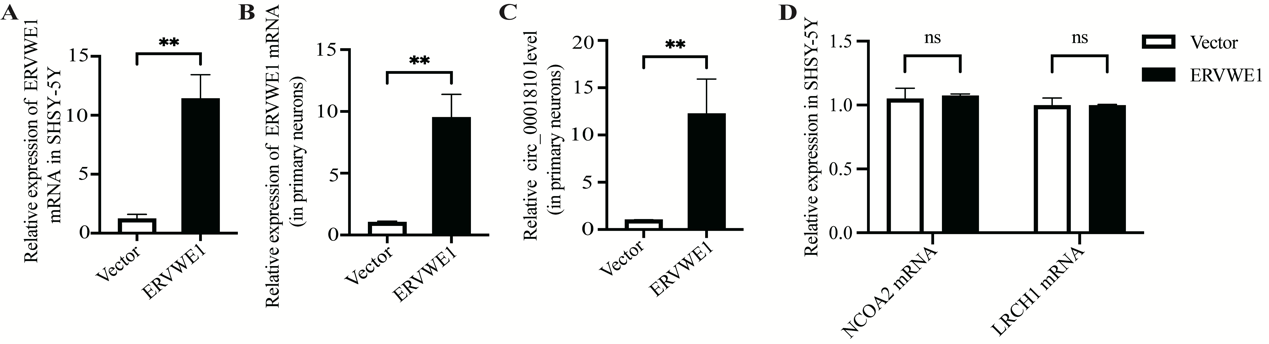


**Fig. S1** The mRNA expression of different genes in cells after ERVWE1 overexpression. (A-B) RT-qPCR detected the ERVWE1 mRNA in SHSY-5Y cells and primary neurons with ERVWE1 transfection. (C) The circ_0001810 level was detected in primary neurons with ERVWE1 transfection. (D) The mRNA expression of NCOA2 and LRCH1 in SHSY-5Y cells after ERVWE1 transfection. Each bar represented the mean ± SD of three independent experiments. ^ns^*p*>0.05, **p*<0.05, ***p*<0.01.


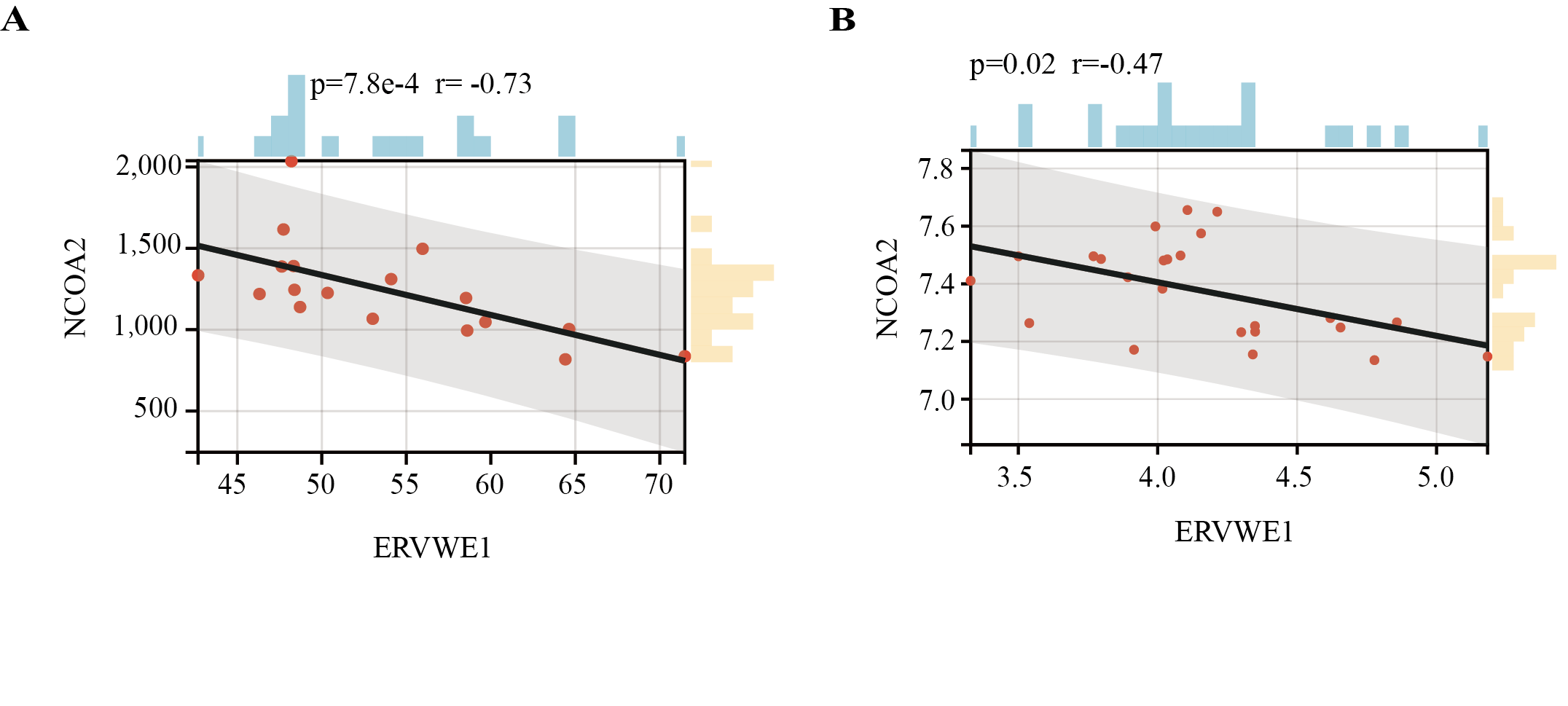


**Fig. S2** Correlation analyses of ERVWE1 and NCOA2 in schizophrenia dataset. (A) GSE53987 dataset. (B) GSE25673 dataset.


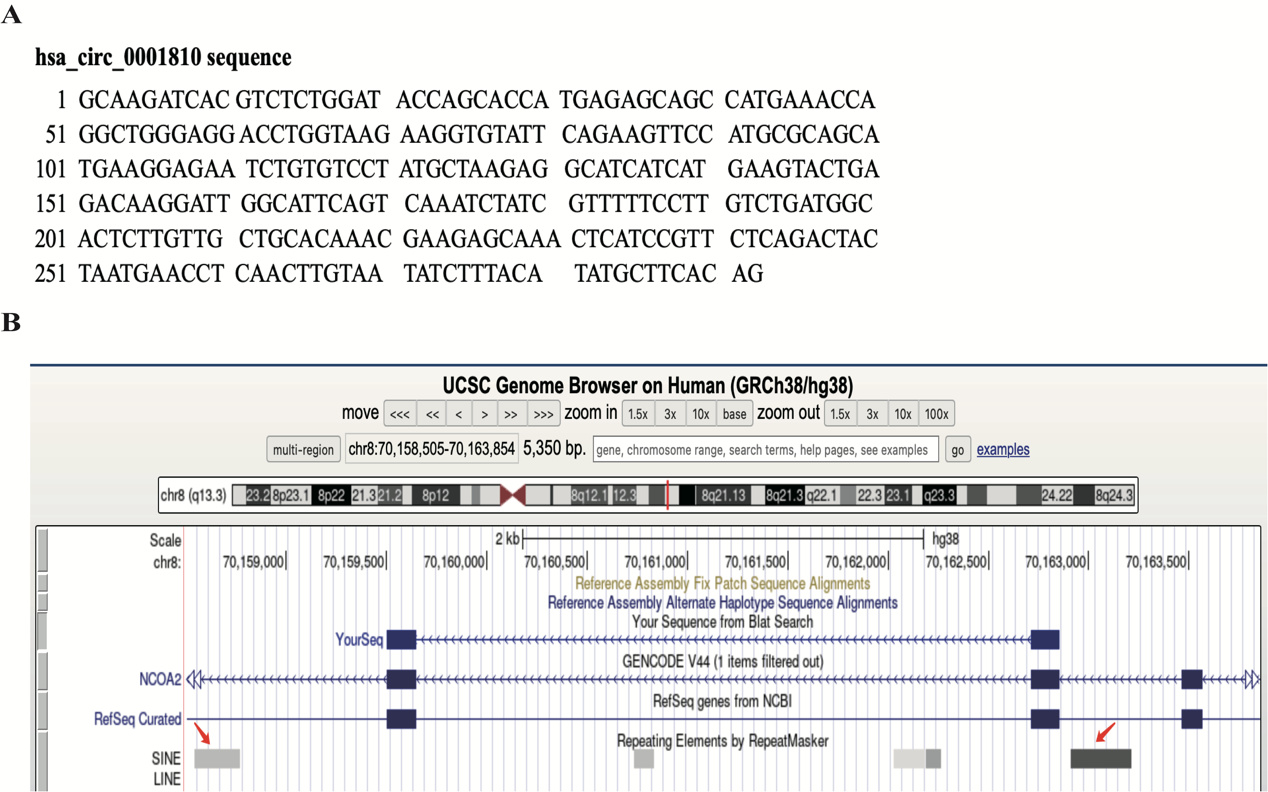


**Fig. S3** The sequence and analysis of circ_0001810. A. The full-length sequence of circ_0001810. B. The SINE analysis of circ_0001810 in UCSC Genome Browser. The red arrow indicated the sequence position of the SINEs.


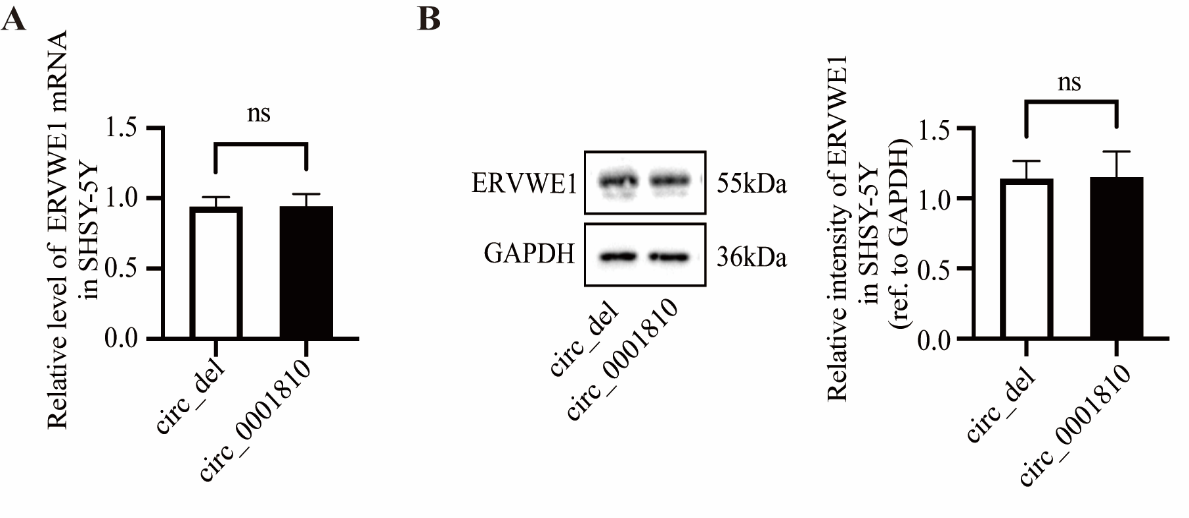


**Fig S4**. The mRNA and protein expression of ERVWE1 in SHSY-5Y cells after circ_0001810 overexpression detected by RT-qPCR and western blots. Results in western blots were quantified using ImageJ and presented as a histogram. Each bar represented the mean ± SD of three independent experiments. ^ns^*p*>0.05.


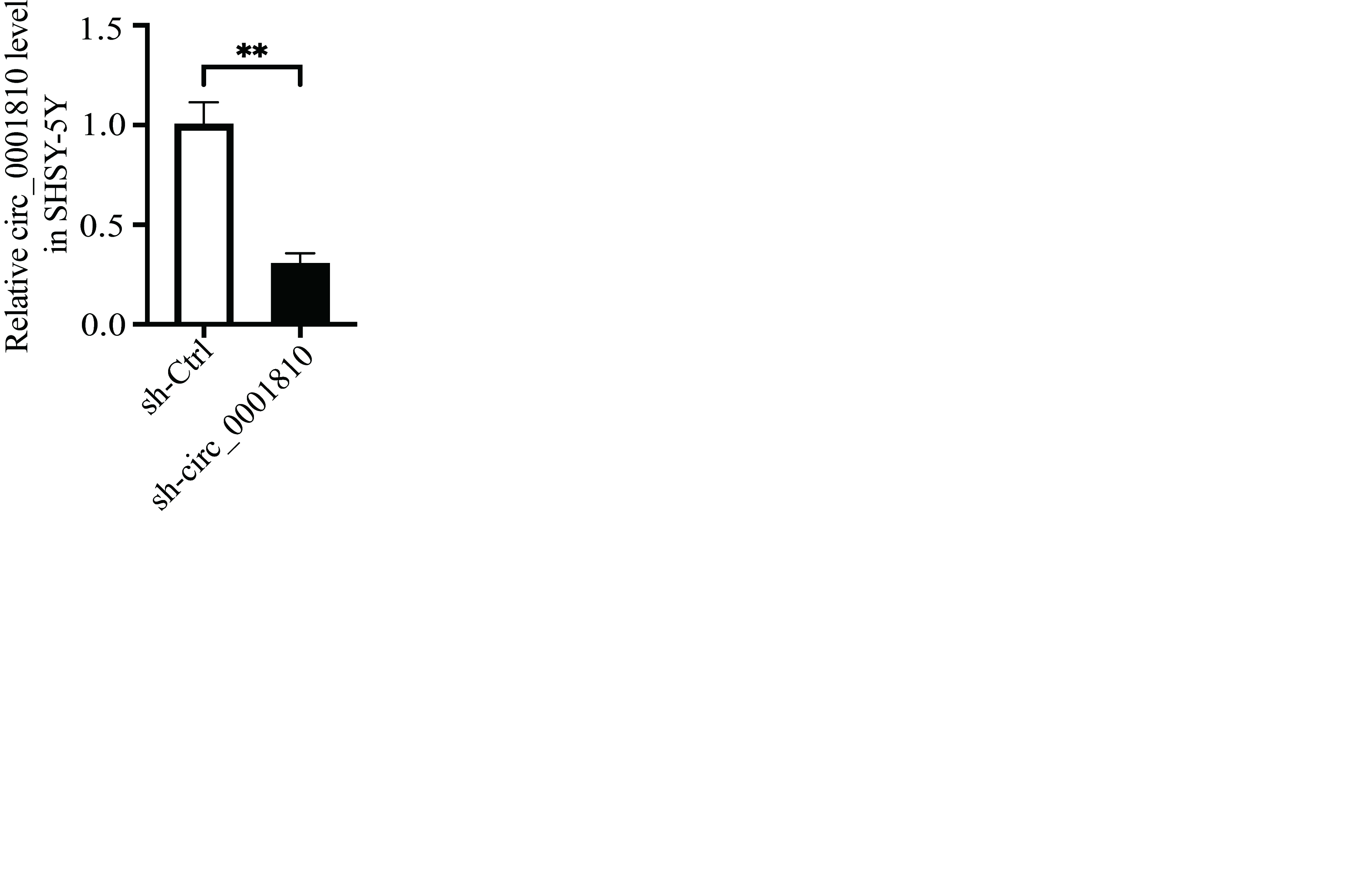


**Fig. S5** RT-qPCR detected the knockdown efficiency of sh-circ_0001810. Ctrl, control. sh, shRNA. Each bar represented the mean ± SD of three independent experiments. ***p*<0.01.


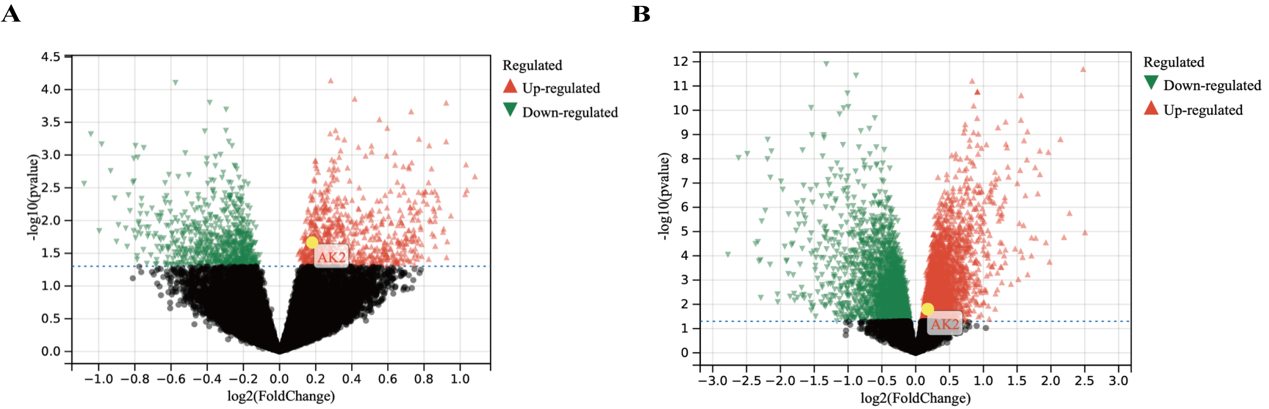


**Fig. S6** The volcano plots of the DEGs in schizophrenia dataset. (A) GSE25673. (B) GSE12649. The highlighted point represented the location of AK2.

**
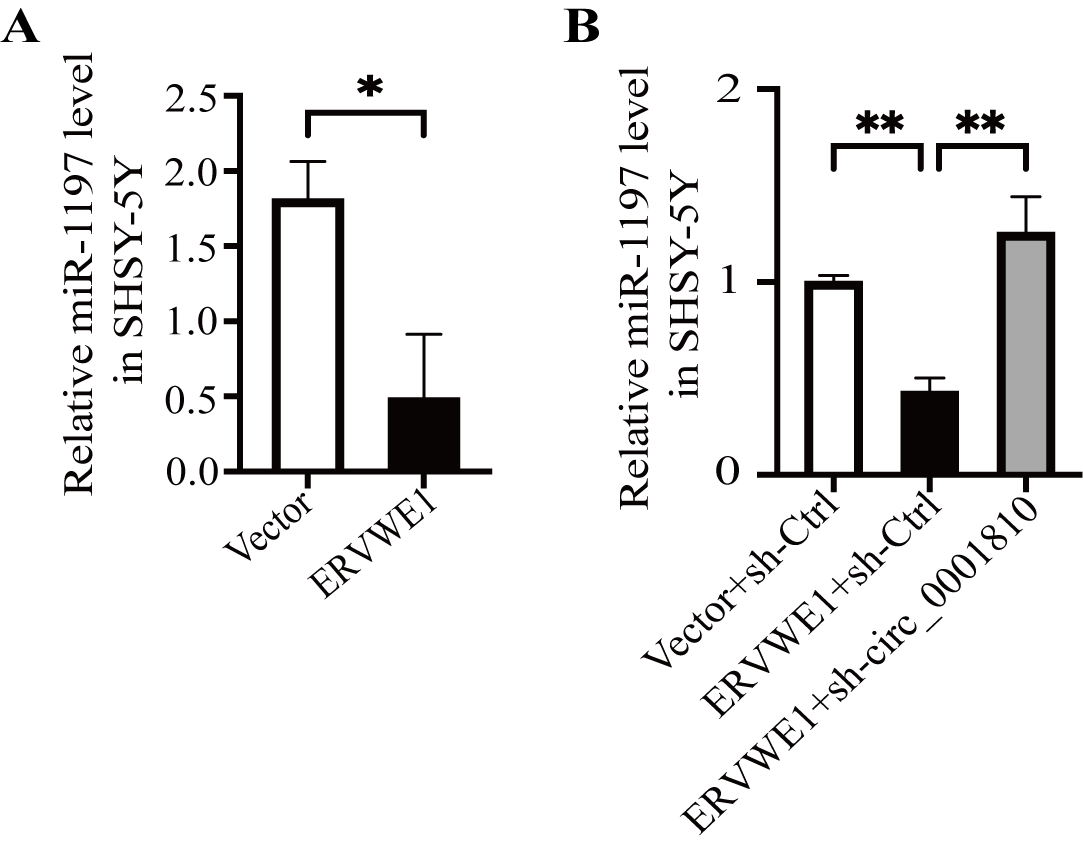
**

**Fig. S7** ERVWE1 inhibited miR-1197 through circ_0001810. (A) RT-qPCR analysis of miR-1197 in SHSY-5Y cells after ERVWE1 overexpression. (B) The miR-1197 level in SHSY-5Y cells with co-transfection ERVWE1 and sh-circ_0001810. Each bar represents the mean ± SD of three independent experiments. **p*<0.05, ***p*<0.01.


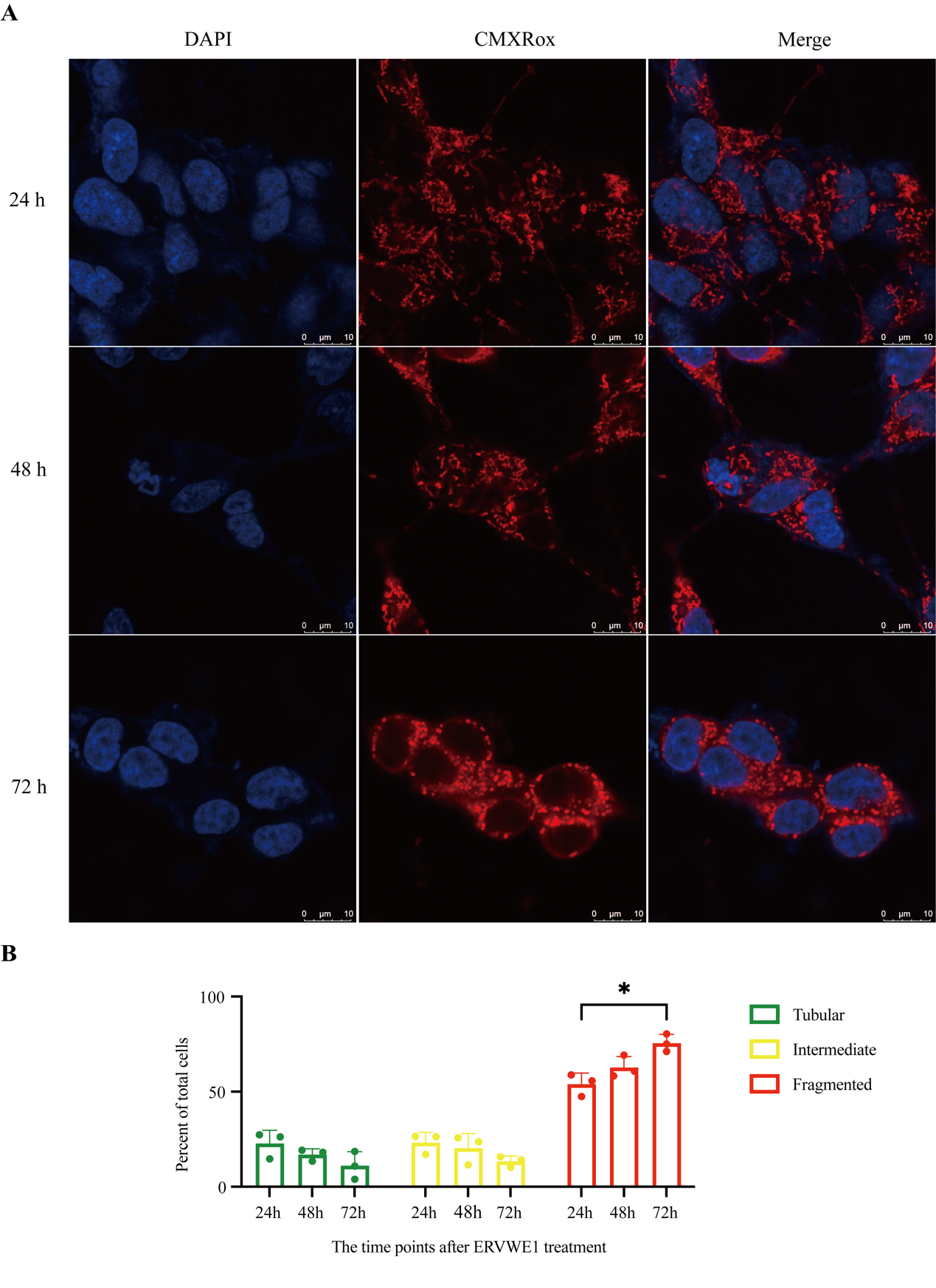


**Fig. S8** Mitochondria fragmentation induced by ERVWE1 in different time points. (A) Mitochondrial morphology in SHSY-5Y cells visualized by MitoTracker Red CMXRos staining after ERVWE1 transfection in 24 hours, 48 hours and 72 hours. (B) Mitochondrial morphology was classified, and the numbers of cells with different morphologies were counted. At least 50 cells per group were counted according to mitochondrial morphologies. Bars represented means ±SD of three independent experiments. **p*<0.05.


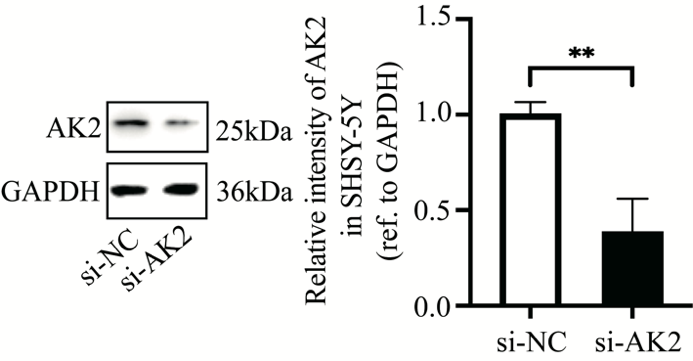


**Fig. S9** The AK2 protein expression after AK2 knockdown in SHSY-5Y cells by western blots. Each bar represents the mean ± SD of three independent experiments. ***p*<0.01.


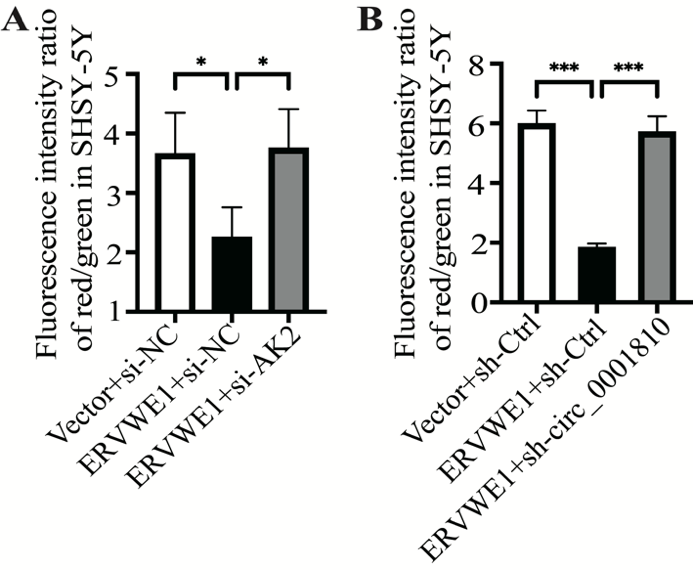


**Fig. S10** Fluorescence intensity ratio of red/green in SHSY-5Y cells. (A) Fluorescence intensity ratio of red/green in SHSY-5Y after co-transfection ERVWE1 and si-AK2. (B) Fluorescence intensity ratio of red/green in SHSY-5Y after co-transfection ERVWE1 and sh_circ_0001810. Each bar represents the mean ± SD of three independent experiments. **p*<0.05, ****p*<0.001.


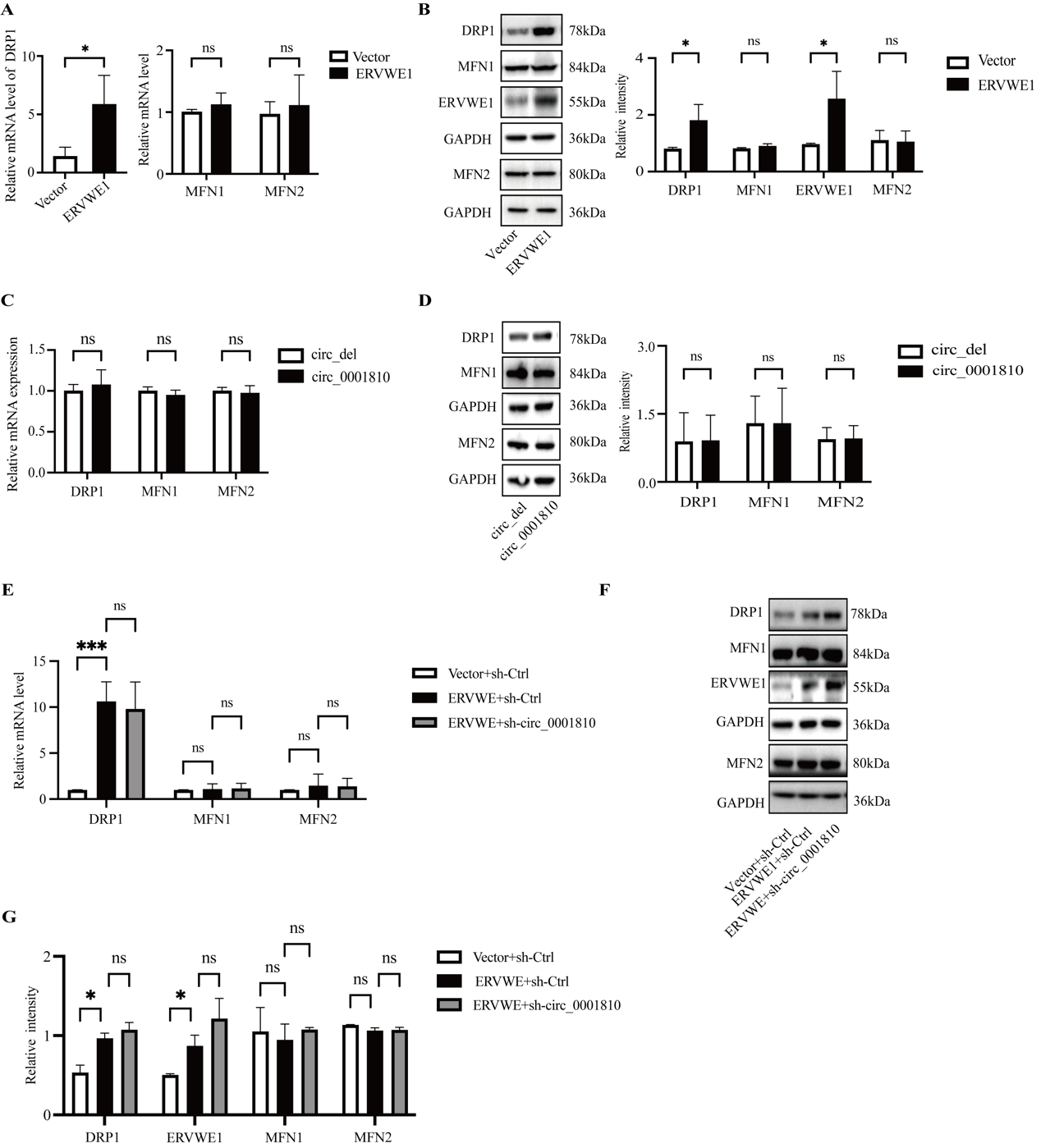


**Fig. S11** The expression levels of DRP1, MFN1, and MFN2 in SHSY-5Y cells. (A-B) RT-qPCR and western blot detected the mRNA and protein expression of DRP1, MFN1, and MFN2 following ERVWE1 overexpression. (C-D) The mRNA and protein expression of DRP1, MFN1, and MFN2 was examined after circ_0001810 overexpression. (E-G) The mRNA and protein expression of DRP1, MFN1, and MFN2 was examined upon co-transfection of ERVWE1 with sh-circ_0001810. Each bar represents the mean ± SD of three independent experiments. ^ns^*p*>0.05, **p*<0.05, *** *p*<0.001.
