# Supplementary material for: Ancestral retrovirus envelope protein ERVWE1 upregulates circ_0001810, a potential biomarker for schizophrenia, and induces neuronal mitochondrial dysfunction via activating AK2
Source: Cell Biosci. 2024 Nov 14;14:138. doi: 10.1186/s13578-024-01318-1 (PMC11566632; doi:10.1186/s13578-024-01318-1)
Supplement: Supplementary file 2 — Additional file 2. [file 13578_2024_1318_MOESM2_ESM.docx]

**Supplementary Figures**


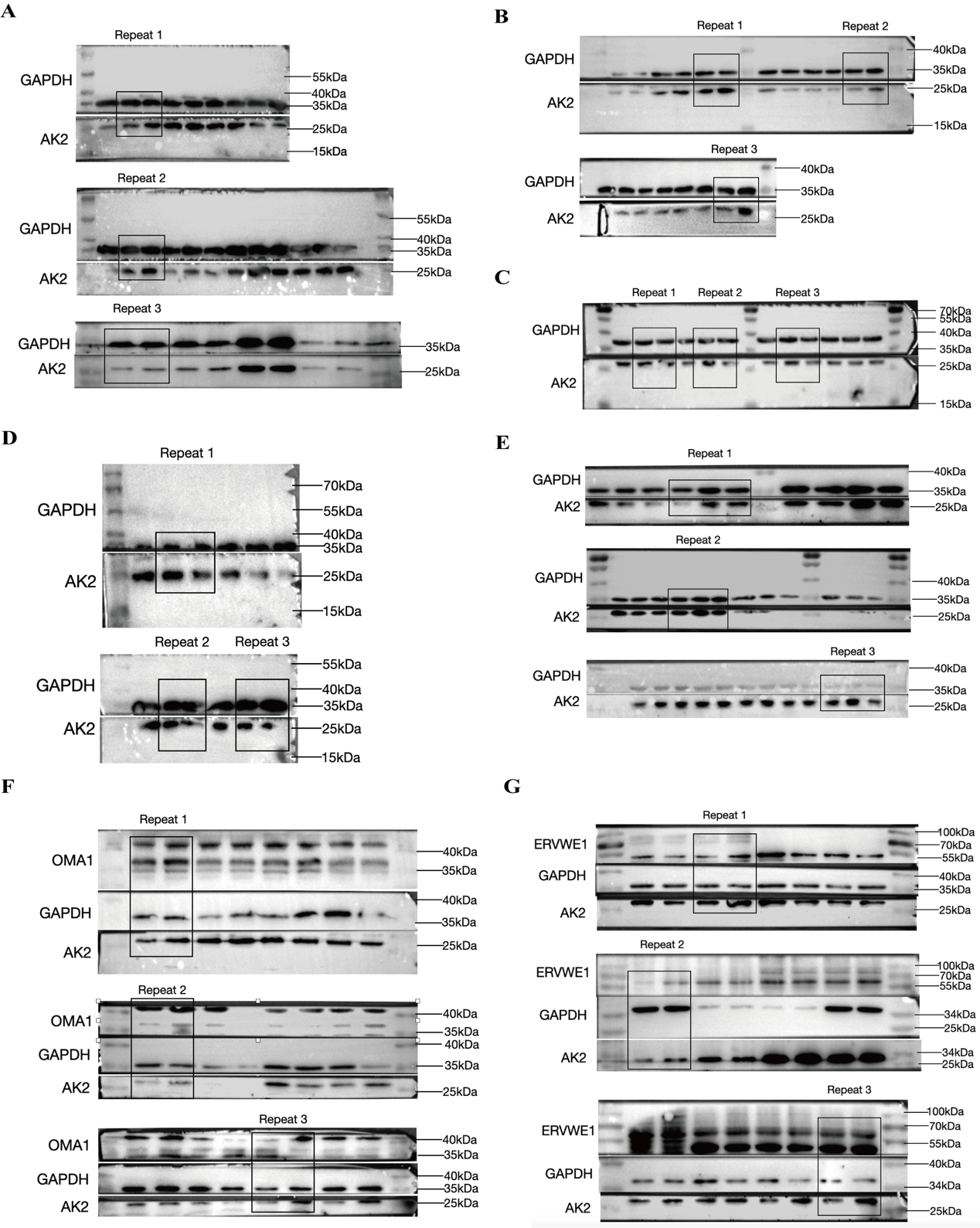


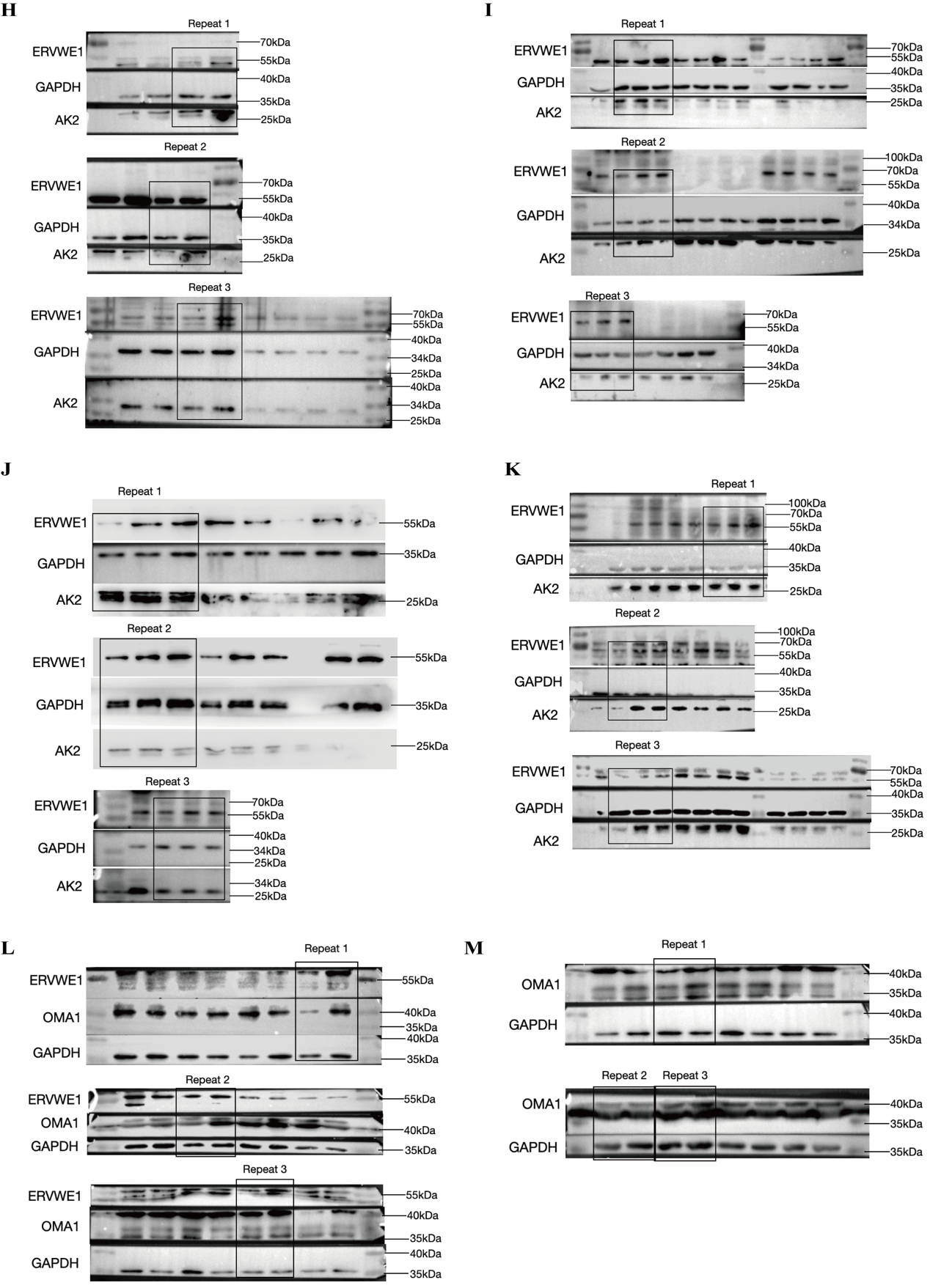


**Fig. S1** Three replicates of each experiment of western blot in the revised manuscript. (A) Relative to Fig. 3G in the revised article. (B) Relative to Fig. 3I in the revised article. (C) Relative to Fig. 3L in the revised article. (D) Relative to Fig. 3O in the revised article. (E) Relative to Fig. 3Q in the revised article. (F) Relative to Fig. 4N in the revised article. (G) Relative to Fig. 5B in the revised article. (H) Relative to Fig. 5C in the revised article. (I) Relative to Fig. 5F in the revised article. (J) Relative to Fig. 5H in the revised article. (K) Relative to Fig. 5J in the revised article. (L) Relative to Fig. 6G in the revised article. (M) Relative to Fig. 7G in the revised article.
